# Livestock host composition rather than land use or climate explains spatial patterns in bluetongue disease in South India

**DOI:** 10.1038/s41598-019-40450-8

**Published:** 2019-03-12

**Authors:** M. M. Chanda, S. Carpenter, G. Prasad, L. Sedda, P. A. Henrys, M. R. Gajendragad, B. V. Purse

**Affiliations:** 1 ICAR-National Institute of Veterinary Epidemiology and Disease Informatics, Bengaluru, India; 2 The Pirbright Institute, Ash Road, Woking, Surrey, GU24 0NF UK; 3 Sardar Vallabhbhai Patel University of Agriculture and Technology, NH 58, Roorkee Road, Modipuram, Meerut, 250110 India; 4 Centre for Health Information, Computation and Statistics (CHICAS), Lancaster Medical School, Faculty of Health and Medicine, Furness Building, Lancaster University, Lancaster, LA1 4YG UK; 5 Centre for Ecology and Hydrology, Lancaster Environment Centre, Bailrigg, Lancaster, LA1 4AP UK; 6 Centre for Ecology and Hydrology, Benson Lane, Crowmarsh Gifford, Oxfordshire, OX10 8BB UK

## Abstract

*Culicoides*-borne arboviruses of livestock impair animal health, livestock production and livelihoods worldwide. As these arboviruses are multi-host, multi-vector systems, predictions to improve targeting of disease control measures require frameworks that quantify the relative impacts of multiple abiotic and biotic factors on disease patterns. We develop such a framework to predict long term (1992–2009) average patterns in bluetongue (BT), caused by bluetongue virus (BTV), in sheep in southern India, where annual BT outbreaks constrain the livelihoods and production of small-holder farmers. In Bayesian spatial general linear mixed models, host factors outperformed landscape and climate factors as predictors of disease patterns, with more BT outbreaks occurring on average in districts with higher densities of susceptible sheep breeds and buffalo. Since buffalo are resistant to clinical signs of BT, this finding suggests they are a source of infection for sympatric susceptible sheep populations. Sero-monitoring is required to understand the role of buffalo in maintaining BTV transmission and whether they must be included in vaccination programs to protect sheep adequately. Landscape factors, namely the coverage of post-flooding, irrigated and rain-fed croplands, had weak positive effects on outbreaks. The intimate links between livestock host, vector composition and agricultural practices in India require further investigation at the landscape scale.

## Introduction

*Culicoides* (Diptera: Ceratopogonidae) are tiny biting flies that transmit arboviruses of livestock, wildlife and humans. These viruses cause diseases that have a significant impact on animal health and welfare, livestock production, trade and livelihoods^1–3^. In the past 20 years, *Culicoides*-borne arboviruses have undergone global changes in epidemiology that have been linked to changes in climate, land use, globalisation of trade and changes in animal husbandry^4^. *Culicoides* are also involved in transmitting human diseases with *Culicoides paraensis* (Goeldi) being the major vector of Oropouche virus (Orthobunyavirus) that causes a febrile illness of people in the neo-tropics^5^. Understanding and predicting how the impacts of *Culicoides*-borne arboviruses vary between geographical areas to inform targeting of control measures^6^ is hampered by their ecological complexity. *Culicoides*, require semi-aquatic developmental sites for egg, larval and pupal development and usually rely on mammalian hosts as a source of blood to produce eggs. Many aspects of this life cycle are affected by variations in temperature, moisture and host and habitat availability^4,7^. In most regions, several *Culicoides* vector species and both wild deer and domestic mammals are involved concurrently in transmission^4,8^ and each of these possesses different climate sensitivities and associations with natural and managed ecosystems^9^.

The emergence of bluetongue virus (BTV: Reoviridae; Orbivirus) and Schmallenberg virus (Bunyaviridae: Orthobunyavirus) in Europe has focussed research effort upon understanding and predicting spatial variability in *Culicoides*-borne arboviruses in relation to environmental drivers^4,10–13^. Research activity has been far less intense in poor tropical and sub-tropical arbovirus endemic zones where selective resistance in livestock tends to be greater, and where vaccines may be used routinely to reduce the impact of what clinical disease occurs^4^. Prior studies of *Culicoides*-borne disease patterns in tropical endemic areas focussed largely on climatic factors, primarily temperature and rainfall^14^. More recent studies have incorporated land use^15^, vegetation^16^ and hosts^17^ as potential explanatory factors, although the relative contribution of these factors to BT impacts are rarely quantified together.

In India between 1997 and 2005 endemic circulation of over 20 different BTV serotypes resulted in over 2000 outbreaks in sheep, involving 0.4 million cases and around 64 000 deaths, making it the top viral cause of disease in this host^18^. In addition to mortality (with local case fatality rates of up to 30%^19^), clinical impacts include weight loss, reductions in wool quality, infertility and lameness. Economic costs include those for veterinary treatment, vaccination, surveillance and trade restrictions^20^. Although there is limited information on breed susceptibility to bluetongue in India^21^, indigenous sheep breeds tend to be asymptomatic despite being widely infected with BTV (high antibody prevalence). Outbreaks were detected largely in exotic (introduced for improvement), or cross-breeds of sheep until the 1980s when cases began to be detected in indigenous sheep^20,22^. These latter cases are thought to have arisen from exotic strains of BTV introduced during breed improvement exercises. Bluetongue mitigation is currently achieved primarily through the use of inactivated pentavalent vaccines^23^, supplied by the state governments of the affected states to small-holder farmers through village veterinary officers.

In common with the transmission of many other arboviral diseases^14,24,25^, rainfall variability during monsoon events is hypothesised to affect the size and timing of BT epidemics in India^20^ and to restrict its spatial distribution primarily to South India which receives high rainfall in both the south west and north east monsoon seasons^26^. However, BT severity varies substantially between districts even within zones subject to similar monsoon conditions, suggesting that factors such as landscape, host and husbandry factors may modulate climate effects on transmission^27,28^ and should be accounted for in an ideal predictive framework. Furthermore, research in Europe has linked vector seasonality and abundance and spread of bluetongue to landscape and host factors alongside climate^29–31^.

The bluetongue system in India is epidemiologically complex. Most of the 26 global BTV serotypes have been detected in India^19,26,32^, and circulate alongside a diverse *Culicoides* fauna that includes at least seven species that have been implicated in arbovirus transmission in other countries (*Culicoides actoni* Smith; *Culicoides brevitarsis* Kieffer; *Culicoides fulvus* Sen and Das Gupta; *Culicoides wadai* Kitaoka; *Culicoides imicola* Kieffer; *Culicoides oxystoma* Kieffer)^33–37^. The immature stages of these species develop in a wide range of semi-aquatic habitats, ranging from animal dung (e.g. buffalo dung: *C. oxystoma*) to organically enriched moist soil (e.g. *C. imicola, C. schultzei, C. peregrinus*). Systematic studies of vectorial capacity and distribution are lacking^33,38^. In the mixed farming systems, practised by the small and marginal farmers of South India, mixed sheep and goat flocks are kept for meat production and indigenous cattle and buffalo maintained for milk production and for draught purposes. Although cattle, goats and buffalo are susceptible to BTV infection and in India are widely infected, displaying high BTV antibody prevalence, they do not usually show clinical disease^20^. Despite this, bovines and some breeds of goats^39^ develop levels of viraemia equivalent to sheep^40^ and can thus theoretically infect *Culicoides* and be involved in the onward transmission of BTV making them potential reservoir hosts. Furthermore, the 14 different sheep breeds present in affected states in South India vary in susceptibility to disease effects^21^. Thus, a high diversity of both susceptible sheep and disease resistant, potential reservoir species for bluetongue virus, such as cattle and buffalo, are kept in the same landscape in South India. Breed and species composition of the livestock population, alongside landscape and climate factors are expected to contribute to patterns in bluetongue disease.

Utilising a substantial dataset of clinical BT outbreaks collected since 1992 by the Indian Council of Agricultural Research’s National Institute for Veterinary Epidemiology and Disease Informatics (NVIEDI), this paper investigates the relative roles of climate, land-use and availability of livestock hosts in driving long-term spatial variation in the severity of BT outbreaks in sheep across districts in South India.

This paper uses spatial Bayesian Generalized Linear Mixed Modelling approaches^41^ with the aim of enhancing current disease management systems in the region. Understanding how multiple environmental factors interact to produce variation in disease severity across districts requires quantitative methods that can deal with collinearity between potential risk factors and spatial dependency in errors. The latter may arise due to intrinsic processes (such as disease spread between districts, represented by the spatial autocorrelation) and extrinsic processes (such as trends or drifts in the response which can be partly or totally explained by environmental covariates)^42^. Bayesian generalized linear mixed models overcome these problems by modelling the spatial dependence as random effects, through a prior distribution^43^. We use a modified version of the Besag-York-Mollie (BYM) model^44^ that quantifies the compromise between spatially structured and spatially unstructured random error variation (to account for overdispersion) as well as fixed effects of environmental predictors^41^.

Our specific aims for South India were to test for associations between the number of disease outbreaks in farmed sheep and:

- coverage of rain-fed cropland and irrigated cropland^45^, that may encompass more abundant semi-aquatic habitat for immature *Culicoides*^46^.
- coverage of open forest types and grassland or shrubland, used by small-holder farmers for grazing
- coverage of closed forest types that are avoided by small-holders for grazing.
- sheep densities, particular of sheep of susceptible exotic and cross-breeds in which cases have been more frequently recorded historically^22^.
- densities of disease-resistant hosts such as cattle and buffalo numbers in which infection may circulate and be sustained silently.

The dependent variable is the average number of villages reporting bluetongue outbreaks in sheep per district from 1992 to 2009 (offset by the total number of villages per district). An outbreak is defined as a village with more than one disease affected sheep in a given transmission year. The village is the epidemiological unit for disease reporting in South India because villages are assumed to be relatively homogenous in animal husbandry and environmental conditions. The analysis was restricted to data from three states of South India in which outbreaks are regularly reported, namely Karnataka (n = 27 districts), Andhra Pradesh (n = 22 districts) and Tamil Nadu (n = 29 districts), with 61 out of the total of 78 constituent districts reporting outbreaks of BT over the 18 year study period (see Fig. S1 for state boundaries and location of the study region within India). We used a hierarchical multi-model approach to infer the relative importance of climate, host and landscape effects in explaining disease patterns (see Methods for full details). The predictors were divided into climate, landscape and host categories (Table 1, Figs S2 to S5). Within each of these categories, all possible predictor combinations were fitted and ranked by Deviance Information Criteria (DIC^47^) to identify a best model with the lowest DIC and a top model set that had DIC values within 2 DIC units of the best model. Predictors that were present (in >70% of top models) and significant (in >50%) of top models were offered to a second stage of model fitting. In this stage, all possible model combinations of the top predictors across climate, host and landscape categories were fitted and ranked by DIC. In the second stage, the frequency with which predictors were present and significant in top set of models was again used to infer their importance in explaining disease patterns.

**Table 1:** Potential landscape, climate and host predictors considered in the analysis of bluetongue outbreaks in sheep in South India with mean values ± standard deviation (s.d.) across districts (see Methods for data sources).

Category	Predictor name (abbreviation)	Description and units of predictor	Mean ± s.d.
Landscape	post-flooding or irrigated croplands (irrig. crop)	Areal coverage per district of post-flooding or irrigated croplands, class 11 from the Globcover 2009 dataset in km^2^	1395 ± 1218
	rainfed croplands (rain crop)	Areal coverage per district of rainfed croplands, class 14 from the Globcover 2009 dataset in km^2^	3440 ± 2691
	mosaic cropland and vegetation (crop-veg mosaic)	Areal coverage per district of mosaic cropland and vegetation (grassland/shrubland/forest mix) with 50–70% cropland, class 20 from the Globcover 2009 dataset in km^2^	1176 ± 1437
	mosaic cropland and vegetation (veg-crop mosaic)	Areal coverage per district of mosaic cropland and vegetation (grassland/shrubland/forest) with 50–70% vegetation, class 30 from the Globcover 2009 dataset in km^2^	297 ± 368
	open broad-leaved deciduous forest (open decid.)	Areal coverage per district of open broad-leaved deciduous forest, class 40 from the Globcover 2009 dataset in km^2^	417 ± 806
	closed broad-leaved deciduous forest (closed decid.)	Areal coverage per district of closed broad-leaved deciduous forest, class 50 from the Globcover 2009 dataset in km^2^	406 ± 589
	urban areas and artificial surfaces (urban)	Areal coverage per district of urban areas and artificial surfaces, class 190 from the Globcover 2009 dataset in km^2^	47 ± 71
Climate	annual rainfall amount (ann_rain)	Average annual rainfall amount per district over the study period in mm	1768 ± 468
	south west monsoon rainfall amount (sw_rain)	Average annual amount of rainfall each year falling in the south west monsoon period (June to September) per district (2004–2010) in mm	955 ± 410
	north east monsoon rainfall amount (ne_rain)	Average annual amount of rainfall each year falling in the north east monsoon period (October to December) per district (2004–2010) in mm	54 ± 51
	mean annual temperature (ann_temp)	Average annual temperature across the pixels in a district (1950–2000) in 0.1 °C	267 ± 18
Host	buffalo (buffalo)	Summed number of animals per district of buffalos from the 2007 livestock census	4.98 ± 0.71
	non-descript sheep (nsheep)	Summed number of animals per district of sheep of non-descript breeds of from the 2007 livestock census	4.78 ± 0.99
	exotic and cross-bred sheep (esheep)	Summed number of animals per district of sheep of exotic and cross-breeds from the 2007 livestock census	2.19 ± 1.65
	indigenous cattle (icattle)	Summed number of animals per district of cattle of indigenous breeds from the 2007 livestock census	5.20 ± 0.57
	cross-bred cattle (cbcattle)	Summed number of animals per district of cattle of cross-breeds from the 2007 livestock census	4.78 ± 0.67
	goats (goats)	Summed densities per district sheep of goats from the 2007 livestock census	5.24 ± 0.69

## Results

**Figure 1:**
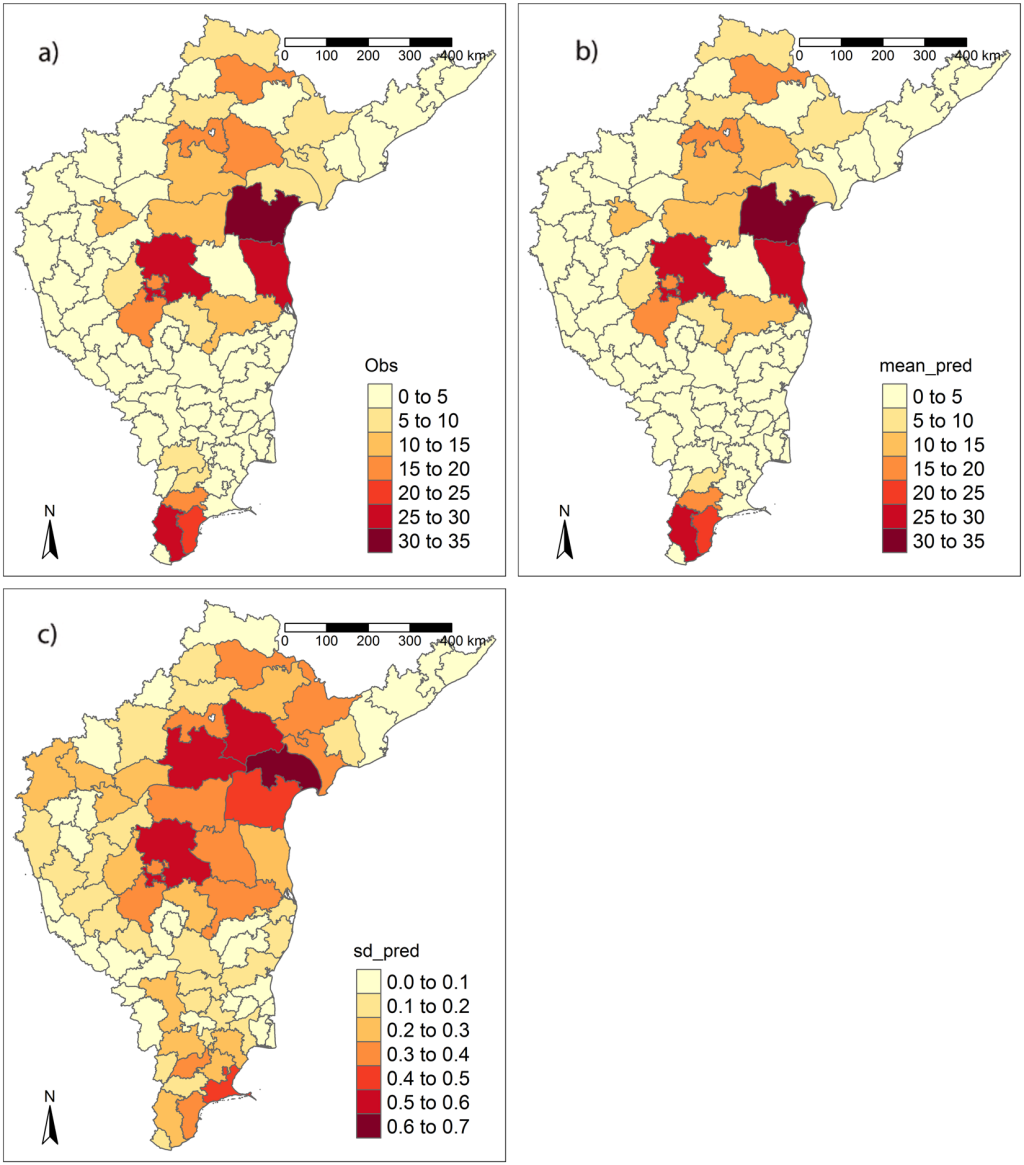
Average annual number of outbreaks affecting districts in India between 1992 and 2009 (**a**) observed data; (**b**) mean prediction per district across top combined environmental models; (**c**) standard deviation of predictions per district across top combined environmental models.

The average annual number of outbreaks ranged from 0 to 33.8 and was highly variable between districts (mean ± s.d. = 4.93 ± 8.10) (Fig. 1a). The average annual number of outbreaks was highest across Andhra Pradesh (particularly in the south of the state) and at the southern tip of India, in Tamil Nadu. Here we first contrast the overall performance and important predictors of the top ranking models in each of the landscape, climate and host categories. Secondly we examine the performance and important predictors of top ranking models that combined predictors across these categories. We also analyse the potential impact of temporal mismatches in our predictor data versus the bluetongue outbreak data on our results.

### Landscape-driven models of BT outbreaks

**Figure 2:**
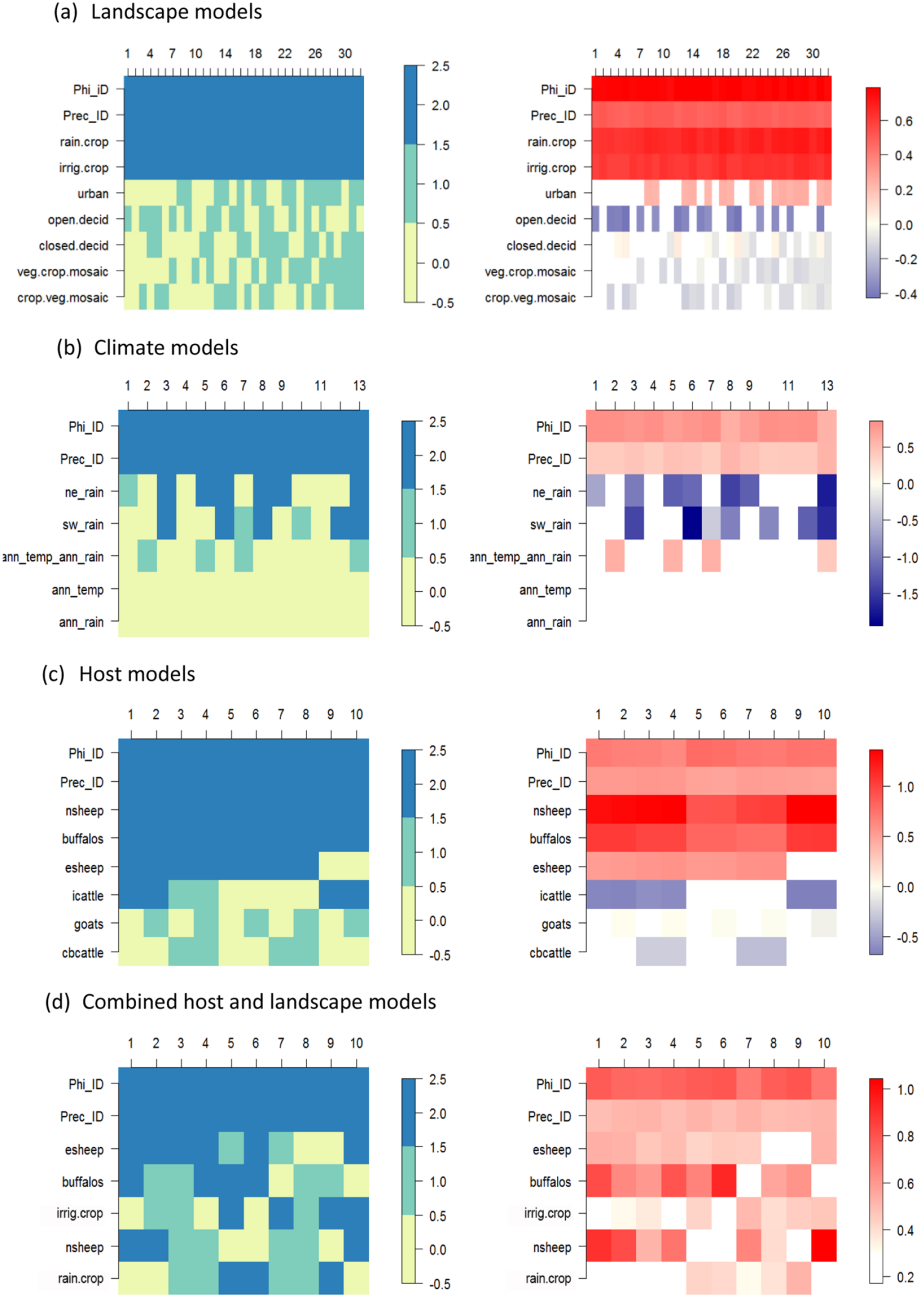
Presence and significance and coefficient values for random and fixed effects present in the top set models of each category. The left hand plots indicate whether predictors are absent (yellow), present but non-significant (green), or present and significant (blue) in each top model. The right hand plots indicates the coefficient values for predictors when present in each top model. In each panel, models are numbered along the x-axis, ranked in order of model performance, from low DIC (“best”, left-hand side) to high DIC (“worst”, right-hand side). The Intercept term was present with significant negative impacts in all models and is not shown. The fixed effect predictors are ordered by the number of times they appeared in the top model set from most (top) to least (bottom) and their names are abbreviated as in Table 1. The spatial random effects are precision (Prec_ID) and the phi parameter, (Phi_ID).

For the landscape suite of predictors, there were 32 models with similar support in the data that were within 2 DIC units of the best model with the lowest DIC (Table S1, DIC = 202.01, Fig. 2a). The best model contained three predictors – cover of post-flooding/irrigated croplands (irrig. crop), cover of rain-fed croplands (Rain crop), and cover of broad-leaved deciduous forest (open decid.). Cover of post-flooding/irrigated croplands and rain-fed croplands had significant (i.e. credible interval of posterior distribution of parameter estimate did not bridge zero) positive effects on mean number of BT outbreaks in all of the top models and produced large increases in DIC when dropped from the best model – 2.36 and 5.44 DIC units respectively. By contrast, cover of broad-leaved deciduous forest was selected in only half of top models, was never significant and caused an increase of only 0.09 DIC units when dropped from the best model, suggesting that this predictor has a weak effect, if any, on BT outbreaks. The remaining landscape predictors never had a significant effect on BT outbreaks (despite each being added to half of the top models, Fig. 2a). Cover of post-flooding/irrigated croplands and rain-fed croplands were the only LANDSCAPE variables offered to the second stage of the environmental variable selection. The best landscape model outperformed the null model substantially (DIC = 210.52, ∆DIC = 8.51).

### Climate-driven models of BT outbreaks

For the climate suite of predictors, there were 13 models with similar support in the data that were within 2 DIC units of the best model with the lowest DIC (Table S2, DIC = 207.08, Fig. 2b). The best performing model contained average North East Monsoon rainfall amount (ne_rain) and average annual rainfall (ann_rain) amount whilst the second best model containing average annual rainfall amount, average annual temperature (ann_temp) and the interaction between these variables had very similar support in the data (∆DIC = 0.31). The best climate model outperformed the null model only marginally (∆DIC = 3.44). None of the climate predictors fulfilled the criteria for being added to the combined environmental model. Although annual rainfall appeared in 10 of the 13 top models and caused an increase of 2.75 DIC units if dropped from the best model, it had a significant (negative) effect on BT outbreaks in only four top models (Fig. 2b). North East Monsoon rainfall was added to seven models but had a significant (negative) effect on BT outbreaks in only six models and produced an increase of only 0.47 DIC units when dropped from the top model. South West Monsoon rainfall was added to seven of the top models and had a significant (negative) effect on BT outbreaks in only five models. Annual temperature was also selected in seven of the top models but had a significant (negative) effect on BT outbreaks in only four models. The annual temperature: annual rainfall interaction was added to four of the top models but was never significant.

### Host-driven models of BT incidence

For the host suite of predictors, there were 10 models with similar support in the data that were within 2 DIC units of the best model with the lowest DIC (Table 2, DIC = 197.63, Fig. 2c). All top models contained significant positive effects of densities of buffalo and non-descript sheep on BT outbreaks and 80% of them contained a significant positive effect of the density of exotic and cross-bred sheep (Fig. 2c). The best model contained the density of buffalo, non-descript sheep, exotic and cross-bred sheep and indigenous cattle and had much better support in the data than the null model (∆DIC = 12.89). Buffalo and non-descript sheep were the most important effects, leading to increases in DIC of 4.18 and 3.92, if dropped from the best model, followed by exotic and cross-bred sheep (∆DIC = 1.96 if dropped from top model). The indigenous cattle variable was added to six of the top models and had a significant negative impact on BT outbreaks in four of these. The effect of indigenous cattle was weak overall however, leading to an increase of only 0.91 DIC units if dropped from the top model. Thus density of buffalos, non-descript sheep and exotic sheep were the only HOST variables offered to the next step of the environmental variable selection.

**Table 2:** Log-likelihood (LL), Deviance Information Criteria (DIC) and effective parameters (pD) for top models of mean BT incidence driven by host predictors.

Fixed effects in model	LL	DIC	pD	∆ DIC	Log-score	RMSE	Proportion of marginal variance explained by spatial effect *ϕ*	
							mean	sd
bt ~ buffalos + nsheep + esheep + icattle	−106.31	197.63	45.60		1.64	0.656	0.69	0.21
bt ~ buffalos + nsheep + esheep + icattle + goats	−111.56	197.92	46.05	0.29	1.70	0.637	0.67	0.22
bt ~ buffalos + nsheep + esheep + icattle + cbcattle	−110.62	198.21	45.93	0.58	1.66	0.650	0.66	0.22
bt ~ buffalos + nsheep + esheep + icattle + cbcattle + goats	−115.87	198.42	46.35	0.79	1.71	0.632	0.64	0.22
bt ~ buffalos + nsheep + esheep	−103.74	198.54	45.49	0.91	1.63	0.656	0.78	0.18
bt ~ buffalos + nsheep + esheep + goats	−109.02	198.79	45.92	1.16	1.67	0.639	0.76	0.19
bt ~ buffalos + nsheep + esheep + cbcattle	−107.73	199.22	45.78	1.59	1.64	0.656	0.72	0.20
bt ~ buffalos + nsheep + esheep + cbcattle + goats	−113.00	199.42	46.20	1.79	1.68	0.638	0.70	0.20
bt ~ buffalos + nsheep + icattle	−104.02	199.59	46.09	1.96	1.65	0.658	0.74	0.18
bt ~ buffalos + nsheep + icattle + goats	−109.21	199.60	46.37	1.97	1.71	0.641	0.74	0.18
*bt ~1	−103.81	210.52	49.11	12.9	1.70	0.625	0.87	0.11

The null intercept-only model* without any covariates is given at the bottom for comparison.

Comparing between best models based on a single suite of predictors, the HOST-driven model (DIC = 197.63) outperformed the LANDSCAPE-driven model substantially (DIC = 202.01, ∆DIC = 4.37), and vastly outperformed the CLIMATE-driven model (DIC = 207.08, ∆DIC = 9.45).

### Combined host- and landscape -driven models of BT outbreaks

All possible combinations of five landscape and host predictors were fitted (irrig. crop, rain crop, buffalo, nsheep, esheep). Of the 33 models fitted, 10 models had similar support in the data, being within 2 DIC units of the best landscape and host model (Table 3, Fig. 2d). The best landscape and host model had a DIC of 198.54, and contained only buffalos, density of non-descript sheep and density of exotic and cross-bred sheep and had slightly less support in the data than the best host model (DIC = 197.63, Table S1, that had also included density of indigenous cattle). Thus combining landscape and host predictors did not improve the ability of models to explain patterns in BT outbreaks compared to models with HOST variables possibly because of the substantial collinearity between landscape and host predictors. Both buffalo (r = 0.650, p < 0.001) and non-descript sheep (r = 0.573, p < 0.001) are positively correlated with post-flooding or irrigated croplands whilst exotic and cross-bred sheep are not (r = −0.170, p = 0.132). Since these combined models perform less well than host only models, we infer the importance of the host predictors from the top host models, namely that densities of buffalos, non-descript sheep and exotic and cross-bred sheep all have consistent and important positive effects on BT outbreaks (Fig. 2c).

**Table 3:** Log-likelihood (LL), Deviance Information Criteria (DIC) and effective parameters (pD) for top models of mean BT incidence driven by a combination of host and landscape predictors.

Fixed effects in model	LL	DIC	pD	∆ DIC	Log-score	RMSE	Proportion of marginal variance explained by spatial effect *ϕ*	
							mean	s.d.
bt ~ buffalos + nsheep + esheep	−103.74	198.54	45.49	0.00	1.63	0.656	0.78	0.18
bt ~ irrig. crop + buffalos + nsheep + esheep	−107.65	198.65	45.63	0.11	1.64	0.655	0.74	0.19
bt ~ irrig. crop + rain crop + buffalos + nsheep + esheep	−111.99	198.87	45.95	0.33	1.65	0.650	0.73	0.19
bt ~ rain crop + buffalos + nsheep + esheep	−108.35	198.93	45.97	0.39	1.65	0.647	0.75	0.18
bt ~ irrig. crop + rain crop + buffalos + esheep	−108.38	199.22	45.41	0.68	1.63	0.664	0.77	0.17
bt ~ rain crop + buffalos + esheep	−105.33	199.94	45.72	1.40	1.64	0.658	0.79	0.16
bt ~ irrig. crop + rain crop + nsheep + esheep	−108.97	200.28	46.47	1.74	1.67	0.653	0.68	0.20
bt ~ irrig. crop + rain crop + buffalos + nsheep	−109.06	200.32	46.20	1.78	1.65	0.657	0.77	0.17
bt ~ irrig. crop + rain crop + buffalos	−105.19	200.37	45.64	1.83	1.64	0.669	0.79	0.15
* bt ~ 1	−103.81	210.52	49.11	11.98	1.70	0.625	0.87	0.11

The null intercept-only model* without any covariates is given at the bottom for comparison.

The overall accuracy of host models was good with low RMSE values in comparison to the observed range of variability in average BT outbreaks, ranging from 0.63 to 0.66 across top models (Table 2, see match between Fig. 1a,b). Out-of-fit model performance was also good, with little evidence that CPO values clustered around 0 for any of the top host models (Fig. S6), and a low logarithmic score for all top host models (Table 2).

In the models based only on hosts, the proportion of marginal variance explained by the spatially structured effect (*ϕ*) ranged from 0.64 to 0.78 (where 1 represents a purely spatial model), indicating a prevalent effect of the spatially structured random effect on average BT outbreak numbers (Table 2). Of the overall variance explained by the top host model for example, the unstructured random effect makes up 18.8%, the spatially-structured random effect 69.4% and the fixed effects 11.8%. Although relatively weak effects, the host covariates are statistically significant in the model, as the credible intervals for corresponding coefficients do not cross zero (Table 4) and the DIC for the null intercept-only model (without fixed effects) is 12.9 DIC units higher than the best model with fixed host and random effects. This is also confirmed by the high Pearson correlation values obtained between observations and predictions when top host models are fitted only with fixed effects but no random effects (Table S3). Thus, these host covariates are a good approximation of the general spatial trend in the data, despite additional noise, with the majority of the departures from this trend due to spatial autocorrelation (probably dependent on the disease transmission process).

**Table 4:** Posterior means, standard deviation and credible intervals for fixed and random* effects in the top host model of bluetongue outbreaks.

Model effect	Posterior mean	s.d.	2.5% quantile	97.5% quantile
(Intercept)	−6.779	0.231	−7.269	−6.363
buffalos	1.039	0.341	0.383	1.725
nsheep	1.279	0.427	0.481	2.165
esheep	0.520	0.230	0.082	0.988
icattle	−0.634	0.315	−1.268	−0.027
Precision*	0.536	0.147	0.303	0.875
Phi*	0.698	0.211	0.2192	0.9737

**Figure 3:**
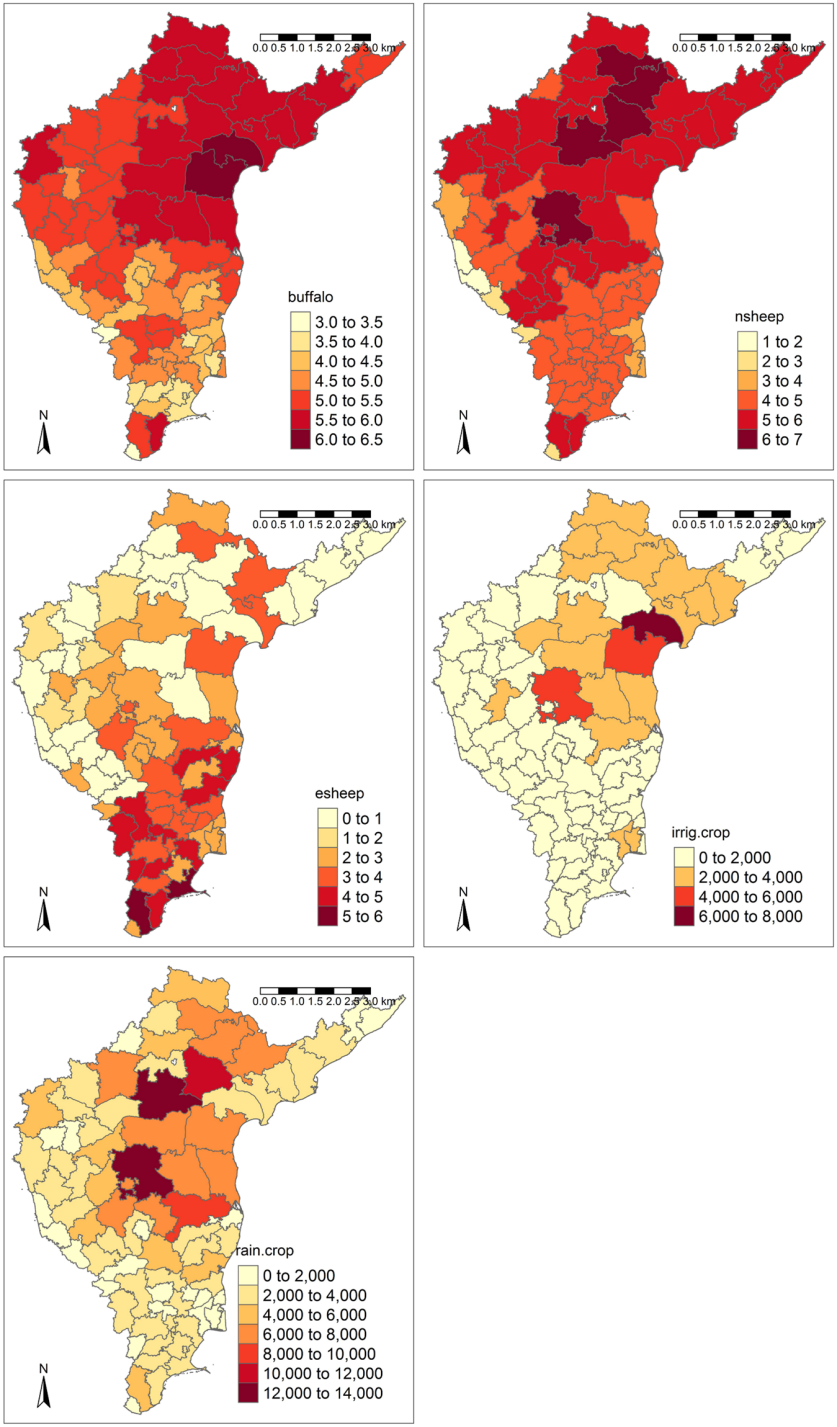
District-level variability in key environmental predictors of average number of BT outbreaks from 1992–2009. (**a**) Buffalo density (buffalos) (**b**) non-descript sheep density (nsheep) (**c**) exotic and crossbred sheep density (esheep) (**d**) post-flooding/irrigated cropland cover (irrig. crop) (**e**) rain-fed cropland cover (rain crop).

In addition to the landscape-host associations mentioned above, across South India densities of non-descript sheep are positively correlated with those of buffalo (r = 0.669, p < 0.001), indigenous cattle (r = 0.545, p < 0.001) and goats (r = 0.447, p < 0.001). Densities of exotic sheep are by contrast negatively correlated with buffalo (r = −0.377, p < 0.001) and indigenous cattle (r = −0.363, p < 0.001), but positively correlated with cross-bred cattle (r = 0.371, p < 0.001). Examining the geographical coincidence of the key environmental predictors of BT outbreaks (Figs 1a and 3), the Andhra Pradesh hot spot of outbreaks is characterised by high densities of buffalo, non-descript sheep and post-flooding or irrigated croplands. In contrast, in Tamil Nadu at the tip of India, the areas of higher BT incidence are characterised by high densities of exotic and crossbred sheep together with high densities of buffalo and non-descript sheep (all exceeding 100,000 head per district), but with very little post-flooding or irrigated cropland. The district values of the spatial random effects are depicted in Fig. S7 are particularly substantial in this southern region of Tamil Nadu, indicating that alternative environmental effects may be constraining outbreaks there.

Considering the potential impacts on our analysis of temporal mismatches in climate predictors and the bluetongue data, very little change in median or range in district-level values of climate predictors was observed between snapshots at the start, middle and end of the study period. Districts have become a fraction warmer (<0.5 °C) on average from the start to the end of the period, and the middle period was drier on average than the start and end of the period (Fig. S8). Most importantly, values of climate predictors were highly correlated between the start, middle and end of the periods at the district level, meaning that districts that started out as warmer or wetter on average, remained warmer or wetter respectively (Table S5). For host predictors, there were substantial changes between livestock census periods (Fig. S9), with the total numbers of sheep and goats per district increasing over the study period, the number of cattle per district decreasing before increasing again to values comparable to the start of the study period, and the number of buffalos per district remaining fairly stable throughout the study period. However, the values of host predictors were highly correlated between livestock census periods at the district level, meaning that districts with more of a particular livestock type at the start of the study period tend to have more of the same livestock type at the end of the study period.

## Discussion

This study advances understanding of the geographical determinants of BT in South India by quantifying the roles of climate, landscape and host factors across a wide geographical area within the same analysis. The resulting models predict average spatial patterns in BT with a high degree of accuracy. We found that host factors, primarily the availability of both susceptible (exotic and cross-bred sheep breeds) and disease-resistant reservoir hosts (buffalo), are more important than land use and climate factors as predictors of variability in BT outbreaks in sheep between districts in South India.

At the national scale, BT is restricted to those parts of India that are heavily affected by monsoons and is linked anecdotally to the timing and intensity of the monsoon seasons^20^. However, our models suggest that within this affected area, spatial variability between districts in long term outbreak numbers is driven by host and landscape heterogeneity rather than by climate. In line with known effects of temperature and moisture on *Culicoides* life cycle parameters (reviewed in^4^) and on the basic reproduction number of bluetongue^48^, many prior studies in tropical and sub-tropical areas have found significant impacts of climatic factors on sub-national patterns of *Culicoides*-borne disease or sero-conversion rates^49^. The direction and magnitude of inferred climate effects, however, varies between regions. For example, in South Africa Baylis *et al*.^24^ found that African horse sickness virus epizootics were associated with the sequence of drought and flood brought by the warm phase of the El Nino Southern Oscillation. In tropical Australia^14^, heavy rainfall during the cyclonic season was fond to be unfavourable for BT transmission, possibly reducing vector population size by destroying breeding sites.

The lack of a detectable climate effect in our study may be because we modelled average patterns in BT outbreaks over a long period of time, potentially blurring important climate-driven inter-annual dynamics (cf. Purse *et al*.^50^) or due to the applied spatial scale units. By averaging patterns across villages within a district we may also miss the local scale effects of landscape factors on the BTV system. We also modelled patterns in outbreak numbers rather than seroconversion rates. Seroconversion rates are directly related to recent infection events and, if related to concurrent environmental conditions, should provide a more accurate picture of the conditions under which transmission occurs. But in India, seroconversion data are collected much less often, and very few places, compared to BT outbreak data^22^, and are thus likely currently to represent a narrow range of the environments in which transmission can occur.

The finding that more outbreaks occur in sheep on average in areas with high buffalo densities is consistent with the high antibody prevalence against BTV observed in this species, without clinical disease, in both India^51^ and China^52^ and indicates this species’ potential importance as a reservoir host. Although the duration and level of BTV viraemia has not been measured in buffalo, isolations have been made from aborted buffalos in India, suggesting a transmissible viraemia^53^. Moreover adult *Culicoides* vectors from Europe are observed to feed preferentially on larger animals such as cattle^54^ or horses^55^. This is explained in terms of their greater body surface but also their greater metabolic weight (calculated as body weight^0.75^) and emission of semio-chemicals including carbon dioxide, that are used by *Culicoides* in host-location. Whether buffalos are preferred over small ruminants as biting hosts for adult *Culicoides* in the Indian setting requires empirical confirmation, because such host preferences have direct consequences for species’ roles in transmission^56^.

Densities of exotic and cross-bred sheep also had a positive effect on outbreak numbers. Such breeds are known to be more susceptible to clinical signs of BT in India^57^ and Nepal^17^ than indigenous local breeds and so infection is more likely to result in a recorded outbreak. Indigenous local sheep breeds in Asia also show antibody prevalence against BT with few disease effects^58,59^, although sporadic clinical cases have been observed in these breeds since the 1980’s^20,60^. The only metric of indigenous sheep density available to our analysis were densities from breeds categorised as non-descript, defined as not having more than 50% similarity to any recognized local breed. These non-descript sheep were found to have a consistent positive association with BT cases. Whether this effect arises because these breeds are disease-resistant and maintain transmission silently or because they are susceptible to disease effects and contribute to recorded cases is difficult to separate given the current lack of empirical studies in India of breed susceptibility. Susceptibility of indigenous breeds in BTV transmission is extremely important to understand given that non-descript sheep constitute a huge proportion of the total sheep population in South India and often co-occur with buffalo and known susceptible sheep breeds (Figs 3, S5). Routine recording and analysis of breed composition for disease cases may also give some insight into their role in transmission.

Although landscape predictors did not improve the description of bluetongue patterns substantially when added to models containing host factors, the landscape-driven models identified important land use types that favour *Culicoides* populations, namely post-flooding/irrigated croplands (irrig. crop) and rain-fed croplands (Rainfed crop), and that are intimately linked to particularly communities of susceptible hosts for bluetongue within agricultural systems in India. The distribution and vectorial capacity of candidate vector species has not been well studied in India^33^ and no quantitative links between BTV-infection or incidence rates and vector community composition have been made. Anecdotally, three key species, namely *C. imicola, C. peregrinus*, and *C. oxystoma* are abundant in BT-affected areas^36,61^. Populations of *C. imicola* and *C. peregrinus*, which both breed in moist and organically enriched soil, have been significantly associated with irrigated areas or other areas of high soil moisture availability (*C. imicola*^62^; *C. peregrinus*^63^). *C. oxystoma*, which develops in a range of habitats including buffalo wallows^64^, has also been found in both active and abandoned rice paddy fields (encompassed by the irrigated cropland class in this study) elsewhere in Asia^46^). It is possible that the extensive rice belt found in Andhra Pradesh (the state most severely affected by BT) makes a substantial contribution to maintaining BT transmission by supporting high, proximate populations of buffalo (Fig. 1c) and *C. oxystoma*.

Landscape or habitat configuration may provide a potential explanation for why buffalos play a stronger role in driving variability in bluetongue outbreaks in sheep compared to cattle in this context. Indigenous cattle were found to have a minor effect and cross-bred cattle no effect on outbreaks compared to buffalos despite the high sero-prevalence of infection detected in cattle in India^51^ and the comparable variation in densities of these species compared to buffalos across districts in the region (Table S4). Whether the body size or herd sizes of buffalos makes them more attractive to host-seeking midges than cattle requires empirical confirmation but buffalos seem to overlap in habitat to a greater extent with susceptible sheep and *C. oxystoma* midges within rice paddies than do cattle. This illustrates the importance of taking a resource or habitat-based approach to predicting the interactions between key hosts and vectors in a disease system^9^.

The inference that buffalos (and potentially non-descript) indigenous breeds of sheep may be playing a key role in BTV transmission has considerable implications for BT control through vaccination in India. Inactivated pentavalent vaccine doses were supplied in 2015 by the respective state governments to small-holder farmers through village veterinary officers. These were targeted only at the susceptible sheep population in endemic districts and cattle and buffalo were not included as vaccination targets. Buffalo outnumber exotic and cross-bred sheep in all but five of the 80 districts in the focal states of Karnataka, Tamil Nadu and Andhra Pradesh, usually by more than two orders of magnitude. This means that partial coverage of the susceptible sheep population is unlikely to achieve herd immunity where-ever these species co-occur. BTV strains could likely attain high levels of transmission in the buffalo population which could act as a source for infection and re-infection of the sheep population. Transmission models from Europe show that vaccinating bovine reservoirs can be an effective strategy for reducing BTV transmission and disease impacts in the sheep population^56^. It is advisable to conduct sero-monitoring of buffalo populations (alongside resistant sheep breeds^26^) in different parts of the country, to modify the composition of the multi-valent inactivated vaccine administered to susceptible sheep accordingly, and to quantify with mathematical models whether and where vaccination of buffalo is needed to reduce transmission of BTV to sheep.

Our models showed good accuracy in both within-sample and out-of-fit tests but a high proportion of the overall variation was attributed to spatially structured random effects (this was particularly true for the districts at the southern tip of India). This indicates that the model could be improved by integration of other unmeasured, spatially structured environmental factors. These could include soil-related parameters (e.g. soil type and water retention capacity), or animal husbandry factors such as dung management and use as fertiliser, local drainage and flooding that influence vector populations or pathogen-related factors. In addition, the land use availability at the start of the study period may have been different due to the conversion of grassland and shrubland to cropland seen across the focal states^65^. Overall, we expect the temporal mismatch between the bluetongue outbreak data (1992–2009) and the rainfall (2004–2010) and host data (2007) to have had limited impact on our results because these conditions, when drawn from coarser resolution data, were highly correlated at district level between the start, middle and end of the study period.

Our approach was to fit a very robust spatial model that carefully analyses the importance of different types of spatial structure alongside climate, land use and hosts in determining bluetongue outbreak patterns as a baseline to inform future space-time model approaches. A properly specified space-time model would have to account for the greater sparsity and lower reliability of disease patterns observed at the monthly level as well as autocorrelation in outbreaks and covariates over time and the possibility that spatial correlation may be structured over time. More-over only the climate covariates are observed on the same monthly time scale as the outbreak data whilst the most epidemiologically relevant host and land use data are available for only a single snapshot in the latter part of the study period. The resulting difficulty in identifying all parameters using a space-time approach would trade-off against determining the relative roles of average climate, land use and host variability in making some areas more susceptible to outbreaks than others. Our framework is highly complementary to the NADRES (National Animal Disease Referral System), the existing early warning system for BT in India, developed and maintained by NIVEDI. NADRES predicts presence rather than outbreak number, quantifies the importance of wide ranging host, landscape and climate drivers but does not account for spatial dependence. To improve targeting of disease control and risk communication, future modelling frameworks for India should investigate the scale-dependent contribution of climate, host and landscape variability to outbreak patterns, over seasonal and inter-annual time-scales, from districts to village level. To optimise control measures that are often taken at the landscape scale, links between particular agricultural ecosystems (like rice paddy cultivation), reservoir and vector community composition and dynamics, and BT impacts should be quantified.

## Methods

### Epidemiological data

District level (Admin-2) annual BT outbreak data (1992–2009) were provided by NIVEDI of the Indian Council of Agricultural Research (ICAR). NIVEDI maintains the livestock diseases database for India, collating outbreak data at district level, based on clinical symptoms observed and reported each month by village-level veterinary officers. Since most goats, cattle and buffalo are disease resistant, reported BT outbreaks are from sheep and thus disease patterns in sheep are analysed here. The village is considered as the epidemiological unit for reporting animal disease outbreaks in India. An outbreak is defined as a village with more than one disease affected sheep in a given transmission year. The analysis was restricted to data from three states of South India in which outbreaks are regularly reported, namely Karnataka (n = 27 districts), Andhra Pradesh (n = 22 districts) and Tamil Nadu (n = 29 districts), with 61 out of the total of 78 constituent districts reporting outbreaks of BT over the 18 year study period (see Fig. S1 for state boundaries and location of the study region within India). The boundaries taken for Andhra Pradesh were those applicable before Telangana state was separated to become an independent state in 2014. District boundaries have changed over the time period for some states, but NIVEDI allocate the past outbreaks to districts according to the most recent district boundaries, prior to providing the data. The dependent variable is the average number of villages reporting bluetongue outbreaks in sheep per district from 1992 to 2009 with total number of villages per district used as the offset (see Modelling Approach) to account for the fact that districts with more villages and more village veterinary officers are likely to report more outbreaks. The city administrative districts of Chennai and Hyderabad were omitted from the analysis as urban districts with little farming they recorded village outbreak data extremely rarely and inconsistently over the study period.

### Environmental data

Landscape: Land use types vary in their suitability for grazing for susceptible and reservoir domestic hosts and in the likely extent of semi-aquatic breeding habitat available to *Culicoides* BTV vectors. The absolute areal coverage in km^2^ of each of seven land use types per district in 2009 were extracted from the Globcover 2009 land-cover map^66^ that has an original pixel resolution of 300 m using Zonal statistics in ArcMap 10.1 (ESRI, Inc., Redlands, CA, U.S.A.) (see Figs S3 and S4). Although the Glob-cover dataset includes additional forest and water-body land use types, these made up less than 3% of the area of districts on average and were therefore unlikely to have been major drivers of overall outbreak numbers.

Host species composition: Densities of the following six host taxa were extracted from the database of National Livestock census data 2007 (http://www.dahd.nic.in/ accessed on 5^th^ May 2014): (1) nondescript indigenous sheep; (2) exotic and cross-bred sheep; (3) goats; (4) crossbred cattle; (5) indigenous cattle and (6) buffaloes. These host density data are collected at village level during the census and then provided as summed district level densities. These predictors were log-transformed (see Fig. S5). Non-descript sheep breeds are indigenous breeds that do not have more than 50% similarity (phenotypically) to any recognized local breed.

Climate variables: *Culicoides* life cycle and BTV transmission cycle parameters are highly sensitive to temperature and moisture availability (reviewed in^4^). The availability of moist soil breeding sites is highly dependent on seasonal rainfall patterns interacting with management factors like dung storage practices, irrigation and drainage. High temperatures increase rates of viral replication, blood digestion, immature midge development and adult biting, but decrease adult survival rates and dry up moist soil breeding sites. Monthly Rainfall Estimates (RFE) were obtained for seven years (2004–2010) from the NOAA/Climate Prediction centre RFE 2.0^67^ at a pixel resolution of 0.1° latitude and longitude and were extracted for districts using zonal statistics ArcMap 10.1 (ESRI, Inc., Redlands, CA, U.S.A.). The average annual total rainfall, south-west monsoon rainfall (months of June to September) and north-east monsoon rainfall (months of October to December) were calculated. The annual mean temperature layer was downloaded from Worldclim^68^ (1950–2000) at a spatial resolution of around 1 km^2^ and extracted and averaged across districts using zonal statistics ArcMap 10.1 (Fig. S2). In addition to these four main effect climate predictors, the interaction between annual rainfall and annual temperature was also considered.

### Modelling approach

All environmental predictors were centred and standardised prior to model-fitting. To take account of spatial autocorrelation, the relationship between the average number of BT outbreaks in sheep per year and environmental predictors was quantified using spatial generalised linear mixed models, implemented in a Bayesian framework.

Let *E*_*i*_ denote the number of villages at risk of BT outbreaks in district *i*(*i* = 1, ….. *n*) used as the offset. The response variable, *y*_*i*_, the average number of BT outbreaks per year (as an integer) over the study period in district *i*, is assumed to follow a Poisson distribution:

$${y}_{i}|{\theta }_{i} \sim Poisson({E}_{i}{\theta }_{i})$$

where *θ*_*i*_ denotes the underlying true area-specific relative risk.

The log risk *η*_*i*_ = log(*θ*_*i*_) was assumed to have the decomposition:

$${\eta }_{i}=\mu +{{z}_{i}}^{{\rm{{\rm T}}}}\beta +{b}_{i}$$

Here *μ* denotes the overall risk level, ${{z}_{i}}^{{\rm{{\rm T}}}}={({z}_{i1},\mathrm{....}.{z}_{ip})}^{{\rm{{\rm T}}}}$ a set of *p* covariates with corresponding regression parameters $\beta ={({\beta }_{1},\mathrm{....}.{\beta }_{p})}^{{\rm{{\rm T}}}}$, and *b*_*i*_ a random effect^41^. The random effects, $b=({b}_{1},\,\,\ldots {b}_{n})$, are used to account for extra-Poisson variation, or spatial dependence between districts due to intrinsic factors or unmeasured abiotic risk factors. Areas that are close in space are assumed to be more similar than areas that are not close, and here districts *i* and *j* were defined as neighbours if they shared a common border, denoted as *i*~*j*.

The area-specific random effect ***b***, was modelled considering a modified Besag-York-Mollie model with a parameterisation suggested by Dean *et al*.^69^

$${\boldsymbol{b}}=\frac{1}{\sqrt{\tau }}(\sqrt{1-\varphi }{\boldsymbol{v}}+\sqrt{\varphi }{{\boldsymbol{u}}}_{\ast }),$$

having covariance matrix

$$\mathrm{Var}({\boldsymbol{b}}|{\tau }_{b})={\tau }_{b}^{-1}((1-\varphi ){\bf{I}}+\varphi {{\bf{Q}}}_{\ast }^{-}).$$

where u_*_ is the scaled spatially structured effect, governed by a Gaussian Markov random field (GMRF) with regions conditionally independent unless classed as neighbours *i*~*j*, Q_*_ is the precision matrix of the Besag model with ${{\bf{Q}}}_{\ast }^{-}$ denoting the generalised inverse and *v*_*i*_ is the unstructured effect modified by Riebler *et al*.^41^ to make the model more intuitive and interpretable. In this parameterisation both *u*_*i*_ and *v*_*i*_ are standardised to have (generalised) variance equal to one. The marginal precision is then *τ* and the proportion of the marginal variance explained by the spatial effect (*u*) is *ϕ*. Employing the hyper-parameterization structure proposed by^41^ allows these hyper-parameters to be mathematically interpretable and not confounded (as in the BYM model). The advantage of this formulation is that the compromise between pure over-dispersion and spatially structured correlation is reflected by the mixing parameter *ϕ*. When *ϕ* = 0, the model reduces to pure over-dispersion, whilst when *ϕ* = 1, the model reduces to the Besag model, i.e. only spatially structured correlation. The model was fitted by integrated nested Laplace approximation using the INLA R package (www.r-inla.org)^70^ and “bym2” model specified inside the model formula. Weak prior distributions were used for *μ* and *β* given by Gaussian distributions with zero mean and precision of 1 × 10^6^. The prior distributions on the standard deviation $1/\sqrt{\tau }$ and *ϕ* are both based on transformed exponential distributions following the penalised complexity framework as described in Simpson *et al*.^71^. Using a transformed exponential distribution has the property that greatest density is at the lowest values for both the precision parameter (*τ*) and the mixing parameter (*ϕ*). This implies that the penalised complexity prior will tend to shrink towards a model of no spatial smoothing in the absence of sufficient support for spatial complexity.

### Model building and selection of environmental predictors

It was not computationally possible to fit all possible combinations (>130,000 combinations) of the 17 main effect predictors and one interaction term. Instead, the main effect predictors were divided into three categories, landscape predictors, climate predictors and host predictors and within each individual category all possible model combinations were first fitted to identify the best predictor variables from that category. Prior to this step, pairs of highly correlated predictors within each category were identified using Pairwise Pearson correlation analyses (r > 0.7, p < 0.001). These pairs were precluded from appearing together in model combinations. Once all models were fitted within a category, the model with the lowest Deviance Information Criteria (DIC)^47^ was identified as the best model. DIC is a generalisation of the Akaike Information Criterion (AIC), and is derived as the mean deviance adjusted for the estimated number of parameters in the model, compromising between model fit and complexity, and providing a measure of out-of-sample predictive error^72^. All models within 2 DIC units of the best model in a category were defined as the top model set, having similar support in the data as the best model, based on the often used rule of thumb of Burnham and Anderson^73^. The number of times that each predictor appeared and had a significant effect on BT outbreaks across the top model set was calculated. Predictors were only passed to the second stage of model selection if they had appeared in over 70% of within-category models, and had significant effects on BT outbreaks in over half of these models.

In the second stage of model selection, all possible combinations of the predictors selected in the first stage were fitted, and the top model set was again identified using DIC. The number of times that each predictor appeared and had a significant effect on BT outbreaks in the top model set was again calculated and mapped against the constituent predictors in the model. This approach reveals whether the effects of individual environmental predictors on BT outbreaks is robust to presence or absence of other predictors in the model and was an attempt to understand the importance of predictors whilst avoiding the drawbacks of model averaging pointed out by Cade^74^.

Considering metrics of model accuracy, we calculated the Root Mean Square Error between the predicted posterior mean values and the corresponding observed annual mean number of BT outbreaks per district. To test the out-of-fit predictive performance of the model, leave-one-out cross validation statistics, namely Conditional Predictive Ordinates (CPOs) were calculated^75^. The CPO expresses the posterior probability of observing the value (or set of values) of *y*_*i*_ when the model is fitted to all data except *y*_*i*_, with a larger value implying a better fit of the model to *y*_*i*_, and very low CPO values suggest that *y*_*i*_ is an outlier and an influential observation. When many CPO values cluster near zero, the model demonstrates poor out-of-fit performance. When many CPO values cluster near one, the model demonstrates good out-of-fit performance^76^. We then calculated the cross-validated logarithmic score^77^, by taking the negative sum of logged CPO values across districts, a lower value of which indicates a better prediction quality of the model.

In the model specification, the generalised variance is rescaled to 1^41^, hence the summed variance across the components was set to be equal to 1. This allowed the contribution of each of the model components (spatially structured and unstructured random effects and the fixed effects) to the overall variance explained to be calculated. This follows a similar approach to that taken by Riebler *et al*.^41^.

However, the default posteriors for the spatial random effect parameters in INLA can result in a bias away from variance being attributed to fixed effects.

To avoid any bias in attributing variance to specific model components, the goodness of fit statistics and the proportion of variance explained by each component was calculated for a set of nested models. For each model in the top models, a full model, including fixed, random and spatial components, was compared with a set of sub-models in which one or two of these components were removed. Thus identifying any bias in the variance attribution by comparison amongst the model set.

### Temporal mismatches in environmental predictors and outbreak data

A potential drawback in our analysis is that while the temperature data reflect the whole period from which outbreak data are drawn, the rainfall, land cover data (from 2009) and the host data (from 2007) are drawn only from the latter half of the period. Datasets that spanned the total period were available for climate and land use. The Indian Meteorological Department New High Spatial Resolution (0.25 × 0.25 degree) Long Period (1901–2013) Daily Gridded Rainfall Data Set Over India^78^, and the ERA Interim daily 2 m temperature (0.25 × 0.25 degree)^79^ were too coarse in resolution compared to the size of a smaller districts (mean district size ± s.d. in km^2^ = 7452 ± 12, range in km^2^ = 178–19223). The alternative longitudinal dataset for land use, the ESA: Land Cover CCI Product^80^, divides the landscape into land use categories that are less explicitly related to midge and host habitats than those in the Globcover products. Similarly, only the more recent livestock census data divide livestock into indigenous and exotic breeds which are known to have different clinical responses to bluetongue (see above).

These temporal mismatches in data will only have a large impact on our analyses of which factors make some districts more susceptible to bluetongue outbreaks in sheep on average, if the late period conditions of climate, hosts and land use are poorly correlated with the equivalent conditions in a district during the earlier parts of the study period. We tested this by performing Pearson’s correlation analysis, paired by district, between the values of climate and host predictors in different snapshots across the study periods.

## Supplementary information

Supplementary information

## Data Availability

The bluetongue data that were analysed during the current study are available from Indian Council of Agricultural Research-National Institute of Veterinary Epidemiology and Disease Informatics (ICAR-NIVEDI) and were obtained from the Director of ICAR-NIVEDI. The livestock data were downloaded from the website of Department of Animal Husbandry, Dairying and Fisheries and were used under licence for this study. The environmental data were compiled from third party sources as referenced in the methods. The bluetongue data are available from the authors on reasonable request, contingent on permission of the Director of ICAR-NIVEDI.

## References

[1] Sinclair M, Bührmann G, Gummow B (2006). An epidemiological investigation of the African Horse Sickness outbreak in the Western Cape province of South Africa in 2004 and its relevance to the current equine export protocol. Journal of the South African Veterinary Association.

[2] Tabachnik WJ (1996). Culicoides variipennis and bluetongue virus epidemiology in the United States. Annual Review of Entomology.

[3] Velthuis AG, Saatkamp HW, Mourits MC, de Koeijer AA, Elbers A (2010). Financial consequences of the Dutch bluetongue serotype 8 epidemics of 2006 and 2007. Preventative Veterinary Medicine.

[4] Purse BV, Carpenter S, Venter GJ, Bellis G, Mullens BA (2015). Bionomics of Temperate and Tropical Culicoides Midges: Knowledge Gaps and Consequences for Transmission of Culicoides-Borne Viruses. Annual Review of Entomology.

[5] Carpenter S, Groschup MH, Garros C, Felippe-Bauer ML, Purse BV (2013). Culicoides biting midges, arboviruses and public health in Europe. Antiviral Research.

[6] Eisen L, Eisen RJ (2011). Using geographic information systems and decision support systems for the prediction, prevention, and control of vector-borne diseases. Annual review of entomology.

[7] Mellor PS, Boorman J, Baylis M (2000). *Culicoides* biting midges: their role as arbovirus vectors. Annual Review of Entomology.

[8] Foxi C (2016). Role of different Culicoides vectors (Diptera: Ceratopogonidae) in bluetongue virus transmission and overwintering in Sardinia (Italy). Parasites & Vectors.

[9] Hartemink N, Vanwambeke SO, Purse BV, Gilbert M, Van Dyck H (2015). Towards a resource-based habitat approach for spatial modelling of vector-borne disease risks. Biological Reviews.

[10] Brand, S. P. C. & Keeling, M. J. The impact of temperature changes on vector- borne disease transmission: Culicoides midges and bluetongue virus. *Journal of the Royal Society Interface* **14**, 10.1098/rsif.2016.0481 (2017). 10.1098/rsif.2016.0481 PMC5378124 28298609

[11] Burgin L, Ekstrom M, Dessai S (2017). Combining dispersion modelling with synoptic patterns to understand the wind-borne transport into the UK of the bluetongue disease vector. International Journal of Biometeorology.

[12] Napp S (2016). Understanding Spatio-Temporal Variability in the Reproduction Ratio of the Bluetongue (BTV-1) Epidemic in Southern Spain (Andalusia) in 2007 Using Epidemic Trees. Plos One.

[13] Sumner, T., Orton, R. J., Green, D. M., Kao, R. R. & Gubbins, S. Quantifying the roles of host movement and vector dispersal in the transmission of vector-borne diseases of livestock. *Plos Computational Biology* **13**, 10.1371/journal.pcbi.1005470 (2017). 10.1371/journal.pcbi.1005470 PMC5393902 28369082

[14] Geoghegan JL, Walker PJ, Duchemin J-B, Jeanne I, Holmes EC (2014). Seasonal Drivers of the Epidemiology of Arthropod-Borne Viruses in Australia. PLOS Neglected Tropical Diseases.

[15] Gao X, Wang HB, Qin HY, Xiao JH (2017). Influence of climate variations on the epidemiology of bluetongue in sheep in Mainland China. Small Ruminant Research.

[16] Klingseisen B, Stevenson M, Corner R (2013). Prediction of Bluetongue virus seropositivity on pastoral properties in northern Australia using remotely sensed bioclimatic variables. Preventive Veterinary Medicine.

[17] Gaire TN (2014). Cross-sectional serosurvey and associated factors of bluetongue virus antibodies presence in small ruminants of Nepal. BMC Research Notes.

[18] Indian Council of Agricultural Research, Project Directorate on Animal Disease Monitoring and Surveillance: Annual Report (IVRI Campus, Hebbal, Bangalore., 2006).

[19] Sreenivasulu D, Subba RM, Reddy Y, Gard G (2003). Overview of bluetongue disease, viruses, vectors, surveillance and unique features: the Indian sub-continent and adjacent regions. Veterinaria italiana.

[20] Prasad, G., Sreenivasulu, D., Singh, K. P., Mertens, P. & Maan, S. In *Bluetongue monograph* (eds Mellor, P. S. Baylis, M. & Mertens, P. P. S.) 167–196 (Elsevier/Academic Press, 2009).

[21] Rao PP (2016). Epidemiology of Bluetongue in India. Transboundary and Emerging Diseases.

[22] Prasad, G., Sreenivasulu, D., Singh, K. P., Mertens, P. P. C. & Maan, S. In *Bluetongue* (eds Mellor, P. Baylis, M. & Merten P. C.) 167–195 (Elsevier Ltd., 2009).

[23] Reddy YK (2010). Development and evaluation of inactivated pentavalent adjuvanted vaccine for bluetongue. Indian Veterinary Journal.

[24] Baylis M, Mellor PS, Meiswinkel R (1998). Horse sickness and ENSO in South Africa. Nature.

[25] Walker AR (1977). Seasonal fluctuations of the *Culicoides* species (Diptera: Ceratopogonidae) in Kenya. Bulletin of Entomological Research.

[26] Rao, P. *et al*. Epidemiology of Bluetongue in India. *Transboundary and emerging diseases* (2014). 10.1111/tbed.12258 25164573

[27] Parham, P. E. *et al*. Climate, environmental and socio-economic change: weighing up the balance in vector-borne disease transmission. *Philosophical Transactions of the Royal Society B: Biological Sciences* **370** (2015). 10.1098/rstb.2013.0551 PMC4342957 25688012

[28] Lambin, E. F., Tran, A., Vanwambeke, S. O., Linard, C. & Soti, V. Pathogenic landscapes: interactions between land, people, disease vectors, and their animal hosts. *Int J Health Geogr* **9** (2010). 10.1186/1476-072X-9-54 PMC2984574 20979609

[29] Pioz M (2012). Why did bluetongue spread the way it did? Environmental factors influencing the velocity of bluetongue virus serotype 8 epizootic wave in France. Plos One.

[30] Purse BV (2012). Impacts of climate, host and landscape factors on *Culicoides* species in Scotland. Medical and Veterinary Entomology.

[31] Rigot T, Vercauteren Drubbel M, Delecolle J, Gilbert M (2013). Farms, pastures and woodlands: the fine-scale distribution of Palearctic *Culicoides spp*. biting midges along an agro-ecological gradient. Medical and Veterinary Entomology.

[32] Maan S (2012). Complete genome sequence analysis of a reference strain of bluetongue virus serotype 16. Journal of virology.

[33] Harrup LE (2016). DNA barcoding and surveillance sampling strategies for Culicoides biting midges (Diptera: Ceratopogonidae) in southern India. Parasites & Vectors.

[34] Udupa, G. *Culicoides spp. (Diptera: Ceratopogonidae) associated with livestock and their relevance to bluetongue infection in Tamil Nadu*. PhD thesis, Tamil Nadu Veterinary and Animal Sciences University (2001).

[35] Dasgupta, S. K. In *Bluetongue: Indian perspective*. (eds Prasad G. & Srivastava, R. N.) 115–188, (CCS HAU Press, 1995).

[36] Reddy CS, Hafeez M (2008). Studies on certain aspects of prevalence of *Culicoides* species. The Indian Journal of Animal Sciences.

[37] Archana M, D’Souza PE, Renuka Prasad C, Byregowda SM (2016). Prevalence of different species of Culicoides in Bangalore rural and urban districts of South India. Journal of Parasitic Diseases: Official Organ of the Indian Society for Parasitology.

[38] Ilango K (2006). Bluetongue virus outbreak in Tamil Nadu, southern India: Need to study the Indian biting midge vectors, Culicoides Latreille (Diptera: Ceratopogonidae). Current Science Bangalore.

[39] Koumbati M, Mangana O, Nomikou K, Mellor PS, Papadopoulos O (1999). Duration of bluetongue viraemia and serological responses in experimentally infected European breeds of sheep and goats. Veterinary Microbiology.

[40] Maclachlan N, Drew C, Darpel K, Worwa G (2009). The pathology and pathogenesis of bluetongue. Journal of comparative pathology.

[41] Riebler A, Sørbye SH, Simpson D, Rue H (2016). An intuitive Bayesian spatial model for disease mapping that accounts for scaling. Statistical Methods in Medical Research.

[42] Dormann CF (2013). Collinearity: a review of methods to deal with it and a simulation study evaluating their performance. Ecography.

[43] Breslow NE, Clayton DG (1993). Approximate inference in generalized linear mixed models. Journal of the American Statistical Association.

[44] Besag J, York J, Mollié A (1991). Bayesian image restoration, with two applications in spatial statistics. Annals of the Institute of Statistical Mathematics.

[45] Diarra M (2015). Modelling the abundances of two major *Culicoides* (Diptera: Ceratopogonidae) species in the Niayes area of Senegal. Plos One.

[46] Yanase T (2013). Molecular identification of field-collected *Culicoides* larvae in the southern part of Japan. Journal of medical entomology.

[47] Spiegelhalter DJ, Best NG, Carlin BP, Van Der Linde A (2002). Bayesian measures of model complexity and fit. Journal of the royal statistical society: Series b (statistical methodology).

[48] Gubbins S, Carpenter S, Baylis M, Wood JLN, Mellor PS (2008). Assessing the risk of bluetongue to UK livestock: uncertainty and sensitivity analyses of a temperature-dependent model for the basic reproduction number. Journal of the Royal Society Interface..

[49] Gao X, Qin H, Xiao J, Wang H (2017). Meteorological conditions and land cover as predictors for the prevalence of Bluetongue virus in the Inner Mongolia Autonomous Region of Mainland China. Preventive Veterinary Medicine.

[50] Purse BV (2004). Predicting the risk of bluetongue through time: climate models of temporal patterns of outbreaks in Israel. Revue Scientifique Et Technique-Office International Des Epizooties.

[51] Kakker N, Prasad G, Srivastava R, Bhatnagar P (2002). Sero-prevalence of bluetongue virus infection in cattle in Haryana, Himachal Pradesh, Punjab and Rajasthan. Indian journal of comparative microbiology, immunology and infectious diseases.

[52] Zhang N, Li Z, Zhang F, Zhu J (2004). Studies on bluetongue disease in thePeople’s Republic of China. Veteriana Italiana.

[53] Chandel BS, Kher HN (1999). Isolation of bluetongue virus from an aborted buffalo foetus of buffalo (*Bubalis bubalis*) in India. Buffalo Bulletin.

[54] Ayllón T (2014). Feeding behaviour of Culicoides spp. (Diptera: Ceratopogonidae) on cattle and sheep in northeast Germany. Parasites & Vectors.

[55] Viennet E (2012). Host preferences of Palaearctic *Culicoides* biting midges: implications for transmission of orbiviruses. Medical and Veterinary Entomology.

[56] Bessell, P. R. *et al*. Impact of temperature, feeding preference and vaccination on Schmallenberg virus transmission in Scotland. *Scientific Reports* **4**, 10.1038/srep05746 (2014). 10.1038/srep05746 PMC4102919 25034464

[57] Lonkar P, Uppal P, Belwal L, Mathur P (1983). Bluetongue in sheep in India. Tropical animal health and production.

[58] Sendow I, Daniels P, Cybinski D, Young P, Ronohardjo P (1991). Antibodies against certain bluetongue and epizootic haemorrhagic disease viral serotypes in Indonesian ruminants. Veterinary microbiology.

[59] Hassan, A., Walton, T. & Osburn, B. In *Bluetongue, African horse sickness, and related orbiviruses: Proceedings of the Second International Symposium*. 155–161 (CRC Press Inc.).

[60] Daniels P, Sendow I, Pritchard L, Eaton B (2003). Regional overview of bluetongue viruses in South-East Asia: viruses, vectors and surveillance. Veterinaria italiana.

[61] Das Gupta SK, Saha NC (1995). A new genus of biting midges (Diptera: Ceratopogonidae) from India. Environment and ecology.

[62] Guichard, S. *et al*. Worldwide niche and future potential distribution of *Culicoides imicola*, a major vector of bluetongue and African horse sickness viruses. *PLoS One* **9**, 10.1371/journal.pone.0112491 (2014). 10.1371/journal.pone.0112491 PMC4229218 25391148

[63] Narladkar, B. W., Deshpande, P. D. & Shivpuje, P. R. Bionomics and life cycle on Culicoides sp (Diptera: Ceratopogonidae). *J Vet Parasitol* **20** (2006).

[64] Wirth, W. W. & Hubert, A. A. The Culicoides of South East Asia (Diptera: Ceratopogonidae). The American Entomological Institute, Gainesville, Florida, 508 pp. (1989).

[65] Tian H, Banger K, Bo T, Dadhwal VK (2014). History of land use in India during 1880–2010: Large-scale transformations reconstructed from satellite data and historical archives. Global and Planetary Change.

[66] Defourny, P. *et al*. GLOBCOVER: a 300 m global land cover product for 2005 using Envisat MERIS time series. In: *ISPRS Commission VII Midterm Symposium* “*Remote Sensing From Pixels to Processes*”, *Enschede, the Netherlands* 8–11. (2006).

[67] Xie, P., Yarosh, Y., Love, T., Janowiak, J. E. & Arkin, P. A. A real-time daily precipitation analysis over South Asia. In: *Preprints of the 16th Conference of Hydrology*, (Orlando, 2002).

[68] Hijmans RJ, Cameron SE, Parra JL, Jones PG, Jarvis A (2005). Very high resolution interpolated climate surfaces for global land areas. International journal of climatology.

[69] Dean C, Ugarte M, Militino A (2001). Detecting interaction between random region and fixed age effects in disease mapping. Biometrics.

[70] Rue H, Martino S, Chopin N (2009). Approximate Bayesian inference for latentGaussian models by using integrated nested Laplace approximations. Journal of the Royal Statistical Society: Series B (Statistical Methodology).

[71] Simpson D, Rue H, Riebler A, Martins TG, Sorbye SH (2017). Penalising Model ComponentComplexity: A Principled, Practical Approach to Constructing Priors. Statist. Sci..

[72] Gelman, A. & Hill, J. *Data analysis using regression and multilevel/hierarchical models*. (Cambridge University Press, 2006).

[73] Burnham, K. P. & Anderson, D. R. *Model Selection and Inference: A Practical Information-Theoretic Approach*. 2nd Edition 74, (Springer-Verlag, New York, 2002).

[74] Cade BS (2015). Model averaging and muddled multimodel inferences. Ecology.

[75] Gelfand, A. E. In *Markov chain Monte Carlo in practice*. 145–161 (Springer, 1996).

[76] Lawson, A. B. *Bayesian disease mapping: hierarchical modeling in spatial epidemiology*. (CRC Press, 2013).

[77] Gneiting T, Raftery AE (2007). Strictly Proper Scoring Rules, Prediction, and Estimation. Journal of the American Statistical Association.

[78] Pai DS (2014). Development of a new high spatial resolution (0.25° X 0.25°) Long period (1901–2010) daily gridded rainfall data set over India and its comparison with existing data sets over the region. MAUSAM.

[79] Srivastava, A. K., Rajeevan, M. & Kshirsagar, S. R. Development of a high resolution daily gridded temperature data set (1969–2005) for the Indian region. *Atmosph. Sci. Lett*., *10*, 249–254. doi:10.1002/asl.232 (2009).

[80] ESA: Land Cover CCI Product User Guide Version 2.0, available at, http://maps.elie.ucl.ac.be/CCI/viewer/download/ESACCI-LC-Ph2-PUGv2_2.0.pdf.

