# Supplementary material for: Livestock host composition rather than land use or climate explains spatial patterns in bluetongue disease in South India
Source: Sci Rep. 2019 Mar 12;9:4229. doi: 10.1038/s41598-019-40450-8 (PMC6414662; doi:10.1038/s41598-019-40450-8)
Supplement: Supplementary file 1 — Supplementary information [file 41598_2019_40450_MOESM1_ESM.pdf]

**Livestock host composition rather than land use or climate explains spatial patterns in bluetongue disease in south India**

M. Chanda, S. Carpenter, G. Prasad, L. Sedda, P. A. Henrys, M.R. Gajendragad and B.V. Purse

Supplementary Information

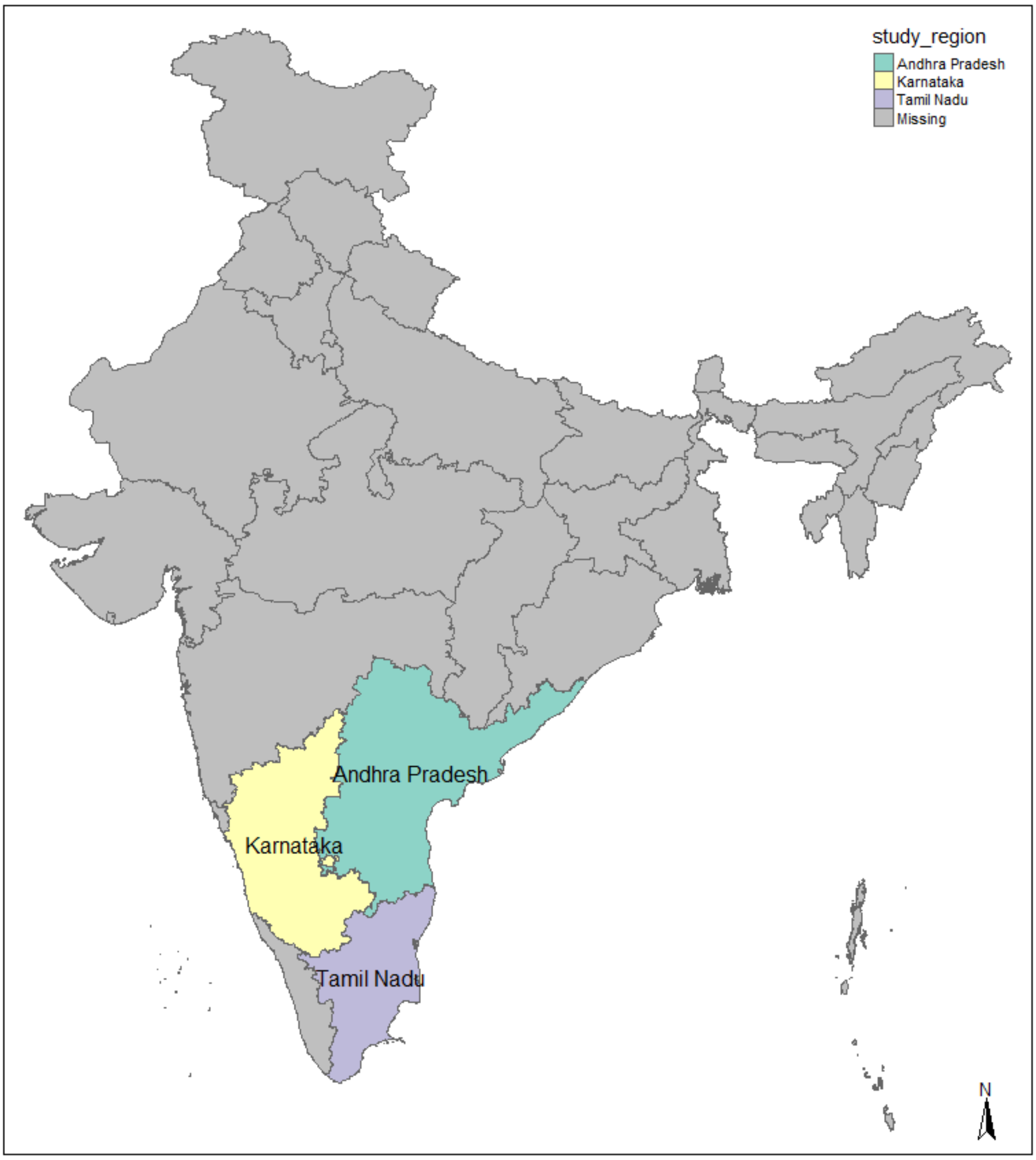


Fig. S1. The location of the study region and consituent study states within India. The boundaries taken for Andhra Pradesh were those applicable before Telangana state was separated to become an independent state in 2014.

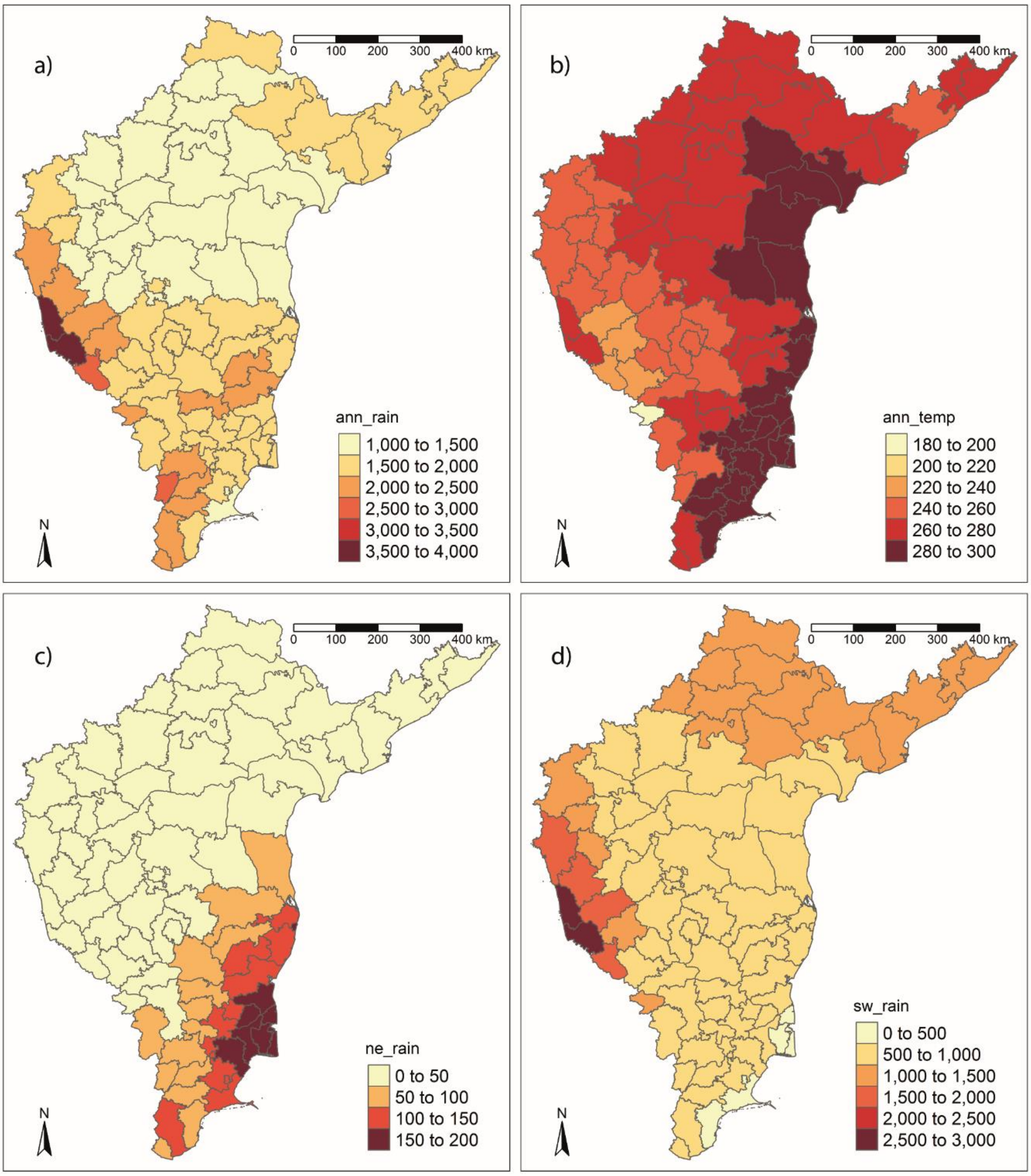


Fig. S2. District-level variability in potential climate predictors of BT outbreaks (a) annual rainfall amount (ann_rain)/mm (b) south west monsoon rainfall amount (sw_rain)/mm (c) north east monsoon rainfall amount (ne_rain)/mm (d) mean annual temperature (ann_temp) in 0.1°C.

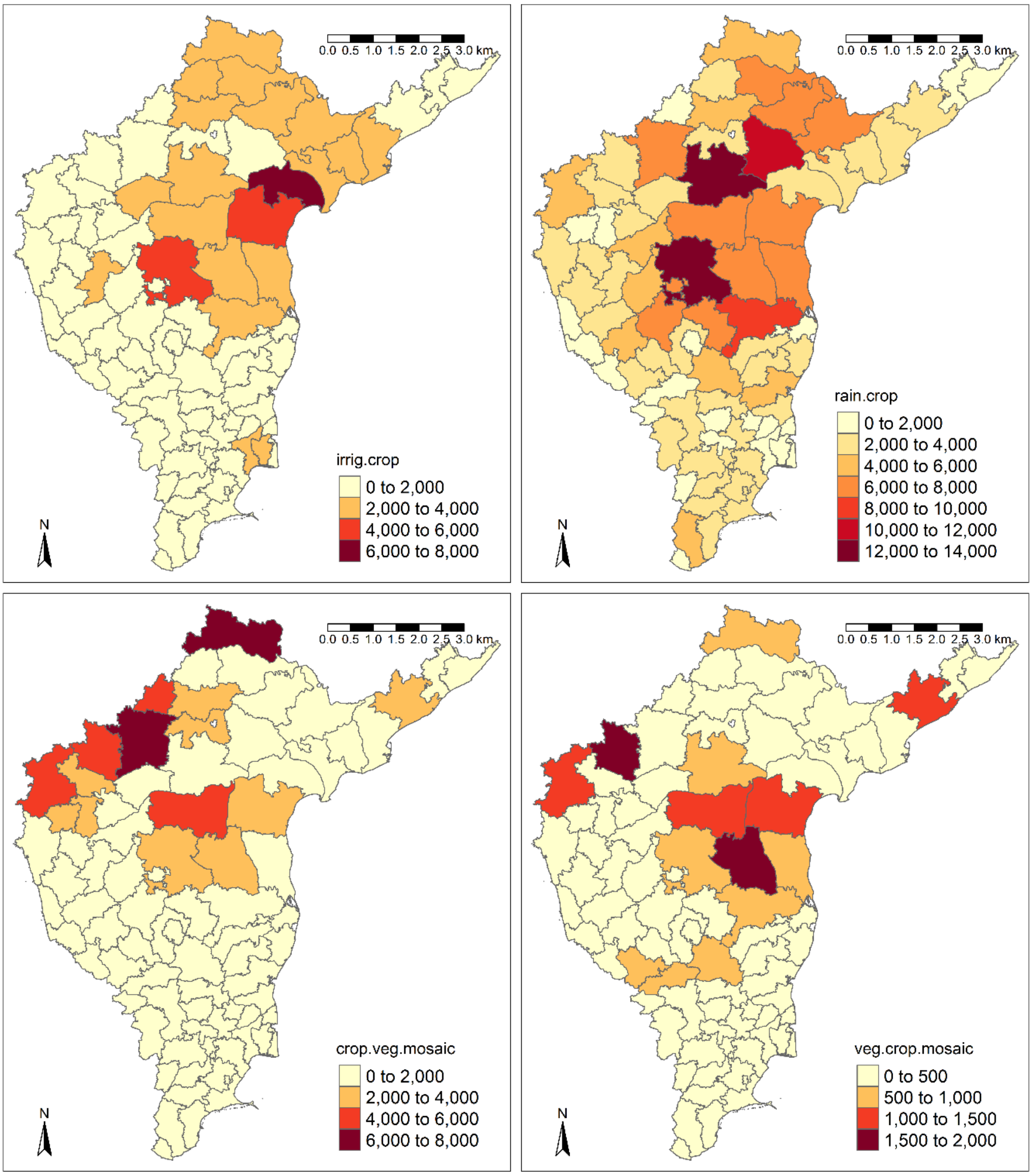


Fig. S3. District-level variability in potential landscape predictors of BT outbreaks, areal coverage in $km^2$ of (a) post-flooding or irrigated croplands (irrig. crop) (b) rainfed croplands (rain crop) (c) mosaic cropland and vegetation (grassland/shrubland/forest) with 50-70% cropland (crop-veg mosaic) (d) mosaic cropland and vegetation (grassland/shrubland/forest) with 50-70% vegatation (veg-crop mosaic)

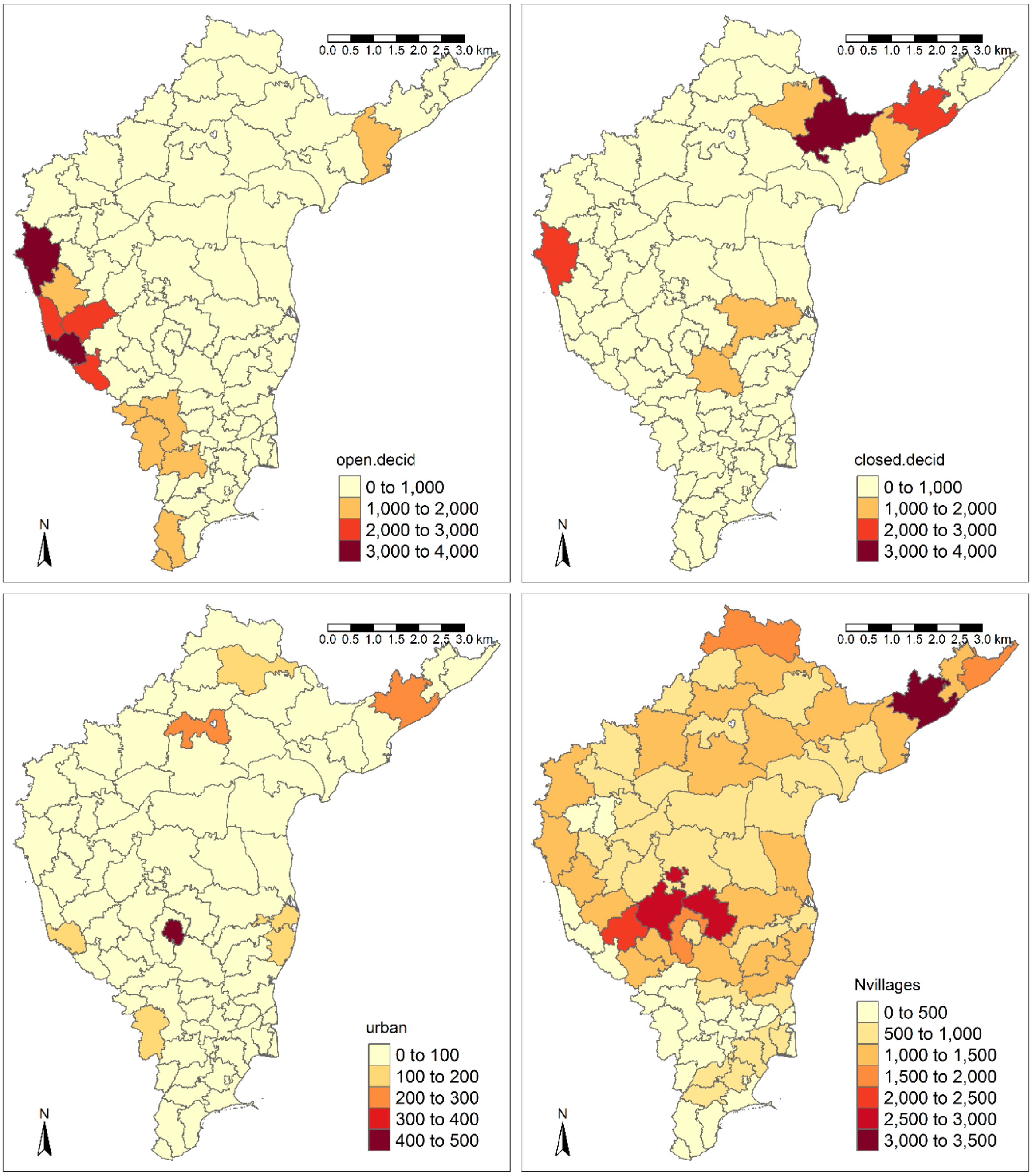


Fig. S4. District-level variability in potential landscape predictors of BT outbreaks, areal coverage in km$^2$ of (a) open broad-leaved deciduous forest (open decid.) (b) closed broad-leaved deciduous forest (closed decid.) (c) urban areas and artificial surfaces (urban), together with (d) the number of villages per district.

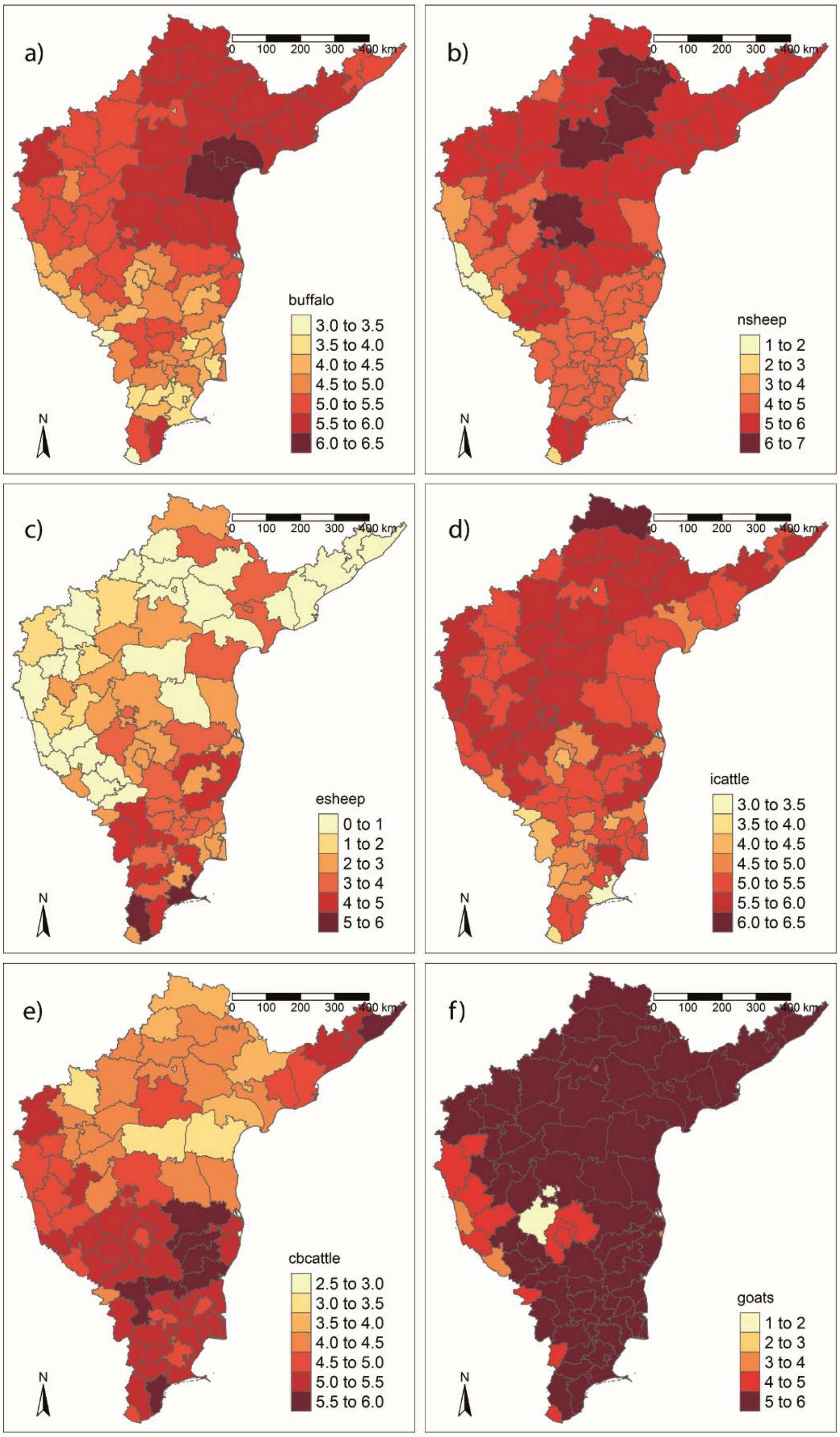


Fig. S5. District-level variability in potential host predictors of BT outbreaks, logged density of (a) buffalos (b) sheep of non-descript breeds (c) sheep of exotic breeds (d) indigenous cattle (e) cross-bred cattle (f) goats.

Table S1. Log-likelihood (LL), Deviance Information Criteria (DIC) and effective parameters (pD) for top models of mean BT incidence driven by the LANDSCAPE suite of predictors.

| Model No. | Fixed effects in model | LL | DIC | Δ DIC | pD | Log-score | RMSE | Proportion of marginal variance explained by spatial effect $\phi$ mean | sd |
|---|---|---|---|---|---|---|---|---|---|
| 31 | bt ~ irrig. crop + rain crop + open decid. | -106.25 | 202.01 | | 46.56 | 1.66 | 0.667 | 0.76 | 0.16 |
| 9 | bt ~ irrig. crop + rain crop | -102.32 | 202.10 | 0.09 | 46.16 | 1.66 | 0.675 | 0.76 | 0.16 |
| 65 | bt ~ irrig. crop + rain crop + crop-veg mosaic + open decid. | -111.03 | 202.18 | 0.17 | 46.89 | 1.68 | 0.657 | 0.77 | 0.16 |
| 69 | bt ~ irrig. crop + rain crop + open decid. + closed decid. | -111.26 | 202.19 | 0.18 | 46.92 | 1.67 | 0.654 | 0.77 | 0.16 |
| 100 | bt ~ irrig. crop + rain crop + crop-veg mosaic + open decid. + closed decid. | -116.01 | 202.31 | 0.30 | 47.23 | 1.69 | 0.644 | 0.78 | 0.16 |
| 30 | bt ~ irrig. crop + rain crop + crop-veg mosaic | -107.23 | 202.40 | 0.39 | 46.54 | 1.67 | 0.664 | 0.76 | 0.16 |
| 70 | bt ~ irrig. crop + rain crop + open decid. + veg-crop mosaic | -111.17 | 202.48 | 0.47 | 46.92 | 1.67 | 0.658 | 0.77 | 0.16 |
| 34 | bt ~ irrig. crop + rain crop + urban | -106.73 | 202.50 | 0.49 | 46.29 | 1.67 | 0.671 | 0.77 | 0.16 |
| 71 | bt ~ irrig. crop + rain crop + open decid. + urban | -110.72 | 202.51 | 0.50 | 46.72 | 1.68 | 0.663 | 0.77 | 0.16 |
| 33 | bt ~ irrig. crop + rain crop + veg-crop mosaic | -107.23 | 202.53 | 0.52 | 46.52 | 1.66 | 0.665 | 0.76 | 0.17 |
| 32 | bt ~ irrig. crop + rain crop + closed decid. | -107.42 | 202.54 | 0.53 | 46.67 | 1.67 | 0.662 | 0.74 | 0.17 |
| 106 | bt ~ irrig. crop + rain crop + open decid. + closed decid. + veg-crop mosaic | -116.11 | 202.55 | 0.54 | 47.24 | 1.68 | 0.644 | 0.78 | 0.16 |
| 102 | bt ~ irrig. crop + rain crop + crop-veg mosaic + open decid. + urban | -115.47 | 202.66 | 0.65 | 47.06 | 1.69 | 0.652 | 0.78 | 0.15 |
| 68 | bt ~ irrig. crop + rain crop + crop-veg mosaic + urban | -111.61 | 202.78 | 0.77 | 46.66 | 1.68 | 0.660 | 0.78 | 0.16 |
| 101 | bt ~ irrig. crop + rain crop + crop-veg mosaic + open decid. + veg-crop mosaic | -115.86 | 202.81 | 0.80 | 47.32 | 1.69 | 0.648 | 0.78 | 0.16 |
| 107 | bt ~ irrig. crop + rain crop + open decid. + closed decid. + urban | -115.72 | 202.82 | 0.81 | 47.12 | 1.69 | 0.652 | 0.78 | 0.16 |
| 66 | bt ~ irrig. crop + rain crop + crop-veg mosaic + closed decid. | -112.32 | 202.86 | 0.85 | 47.05 | 1.69 | 0.652 | 0.75 | 0.17 |
| 74 | bt ~ irrig. crop + rain crop + veg-crop mosaic + urban | -111.57 | 202.91 | 0.90 | 46.61 | 1.67 | 0.662 | 0.77 | 0.16 |
| 122 | bt ~ irrig. crop + rain crop + crop-veg mosaic + open decid. + closed decid. + urban | -120.46 | 202.92 | 0.91 | 47.43 | 1.70 | 0.641 | 0.79 | 0.16 |
| 121 | bt ~ irrig. crop + rain crop + crop-veg mosaic + open decid. + closed decid. + veg-crop mosaic | -120.82 | 202.94 | 0.93 | 47.70 | 1.70 | 0.633 | 0.78 | 0.16 |
| 72 | bt ~ irrig. crop + rain crop + closed decid. + veg-crop mosaic | -112.32 | 202.95 | 0.94 | 47.02 | 1.68 | 0.652 | 0.75 | 0.17 |
| 73 | bt ~ irrig. crop + rain crop + closed decid. + urban | -111.75 | 202.96 | 0.95 | 46.78 | 1.69 | 0.660 | 0.75 | 0.16 |
| 108 | bt ~ irrig. crop + rain crop + open decid. + veg-crop mosaic + urban | -115.61 | 202.97 | 0.96 | 47.08 | 1.68 | 0.653 | 0.77 | 0.16 |
| 67 | bt ~ irrig. crop + rain crop + crop-veg mosaic + veg-crop mosaic | -112.06 | 203.01 | 1.00 | 46.99 | 1.69 | 0.654 | 0.76 | 0.17 |
| 125 | bt ~ irrig. crop + rain crop + open decid. + closed decid. + veg-crop mosaic + urban | -120.55 | 203.16 | 1.15 | 47.43 | 1.69 | 0.642 | 0.78 | 0.16 |

| | | | | | | | | | |
|---|---|---|---|---|---|---|---|---|---|
| 104 | bt ~ irrig. crop + rain crop + crop-veg mosaic + closed decid. + urban | -116.63 | 203.25 | 1.24 | 47.16 | 1.70 | 0.649 | 0.76 | 0.16 |
| 123 | bt ~ irrig. crop + rain crop + crop-veg mosaic + open decid. + veg-crop mosaic + urban | -120.31 | 203.33 | 1.32 | 47.50 | 1.71 | 0.643 | 0.78 | 0.15 |
| 109 | bt ~ irrig. crop + rain crop + closed decid. + veg-crop mosaic + urban | -116.63 | 203.35 | 1.34 | 47.12 | 1.69 | 0.650 | 0.76 | 0.16 |
| 105 | bt ~ irrig. crop + rain crop + crop-veg mosaic + veg-crop mosaic + urban | -116.41 | 203.39 | 1.38 | 47.09 | 1.70 | 0.650 | 0.77 | 0.16 |
| 103 | bt ~ irrig. crop + rain crop + crop-veg mosaic + closed decid. + veg-crop mosaic | -117.12 | 203.46 | 1.45 | 47.50 | 1.71 | 0.641 | 0.74 | 0.17 |
| 128 | bt ~ irrig. crop + rain crop + crop-veg mosaic + open decid. + closed decid. + veg-crop mosaic + urban | -125.26 | 203.54 | 1.53 | 47.90 | 1.72 | 0.630 | 0.78 | 0.16 |
| 124 | bt ~ irrig. crop + rain crop + crop-veg mosaic + closed decid. + veg-crop mosaic + urban | -121.42 | 203.85 | 1.84 | 47.61 | 1.72 | 0.638 | 0.75 | 0.16 |

Table S2. Log-likelihood (LL), Deviance Information Criteria (DIC) and effective parameters (pD) for top models of mean BT incidence driven by the CLIMATE suite of predictors.

| Model No. | Fixed effects in model | LL | DIC | Δ DIC | pD | Log-score | RMSE | Proportion of marginal variance explained by spatial effect $\phi$ mean | sd |
|---|---|---|---|---|---|---|---|---|---|
| 11 | bt ~ ne_rain + ann_rain | -107.45 | 207.08 | | 48.77 | 1.70 | 0.627 | 0.85 | 0.12 |
| 26 | bt ~ ann_rain + ann_temp + ann_temp:ann_rain | -111.25 | 207.39 | 0.31 | 48.96 | 1.70 | 0.621 | 0.83 | 0.14 |
| 7 | bt ~ sw_rain + ne_rain | -105.20 | 207.53 | 0.45 | 48.12 | 1.70 | 0.652 | 0.79 | 0.15 |
| 4 | bt ~ ann_rain | -104.26 | 207.55 | 0.47 | 48.90 | 1.70 | 0.631 | 0.84 | 0.13 |
| 31 | bt ~ ne_rain + ann_rain + ann_temp + ann_temp:ann_rain | -112.30 | 207.65 | 0.57 | 48.58 | 1.73 | 0.646 | 0.74 | 0.18 |
| 17 | bt ~ sw_rain + ne_rain + ann_rain | -108.75 | 208.10 | 1.02 | 48.27 | 1.71 | 0.649 | 0.79 | 0.15 |
| 30 | bt ~ sw_rain + ann_rain + ann_temp + ann_temp:ann_rain | -114.69 | 208.24 | 1.16 | 49.26 | 1.71 | 0.616 | 0.84 | 0.14 |
| 18 | bt ~ sw_rain + ne_rain + ann_temp | -106.95 | 208.29 | 1.21 | 48.30 | 1.74 | 0.661 | 0.60 | 0.21 |
| 23 | bt ~ ne_rain + ann_rain + ann_temp | -109.02 | 208.33 | 1.25 | 48.69 | 1.73 | 0.653 | 0.73 | 0.18 |
| 8 | bt ~ sw_rain + ann_rain | -107.29 | 208.33 | 1.25 | 49.03 | 1.70 | 0.632 | 0.83 | 0.14 |
| 14 | bt ~ ann_rain + ann_temp | -108.05 | 208.57 | 1.49 | 49.22 | 1.72 | 0.631 | 0.81 | 0.15 |
| 2 | bt ~ sw_rain | -103.58 | 208.59 | 1.51 | 48.79 | 1.70 | 0.642 | 0.83 | 0.14 |
| 32 | bt ~ sw_rain + ne_rain + ann_rain + ann_temp + ann_temp:ann_rain | -113.94 | 209.00 | 1.92 | 48.62 | 1.75 | 0.651 | 0.57 | 0.23 |

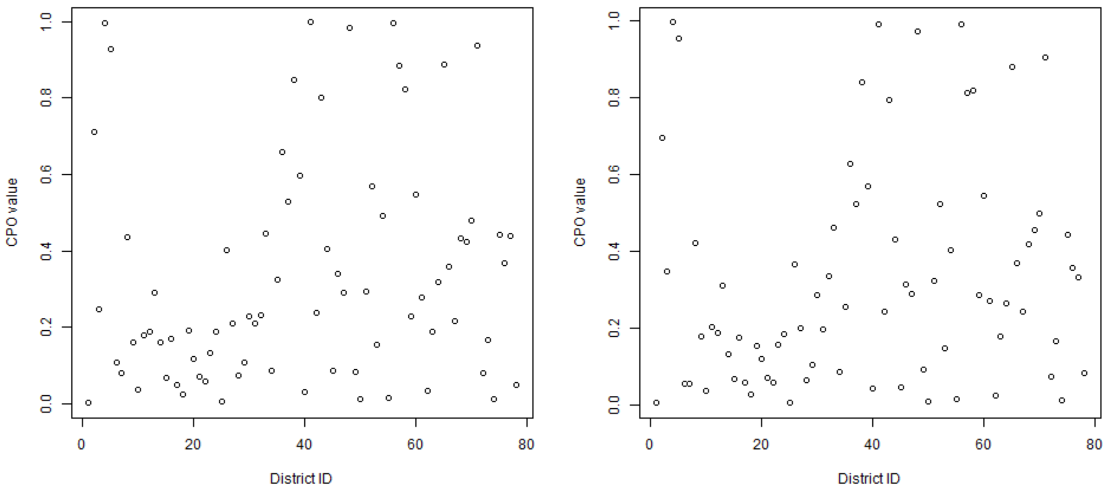


Fig. S6. Conditional predictive ordinate values for districts when mean number of outbreaks per year are predicted from (a) the top host model and (b) the top combined host and landscape model. When many CPO values cluster near zero, the model demonstrates poor out-of-fit performance. When many CPO values cluster near one, the model demonstrates good out-of-fit performance (Lawson, 2009).

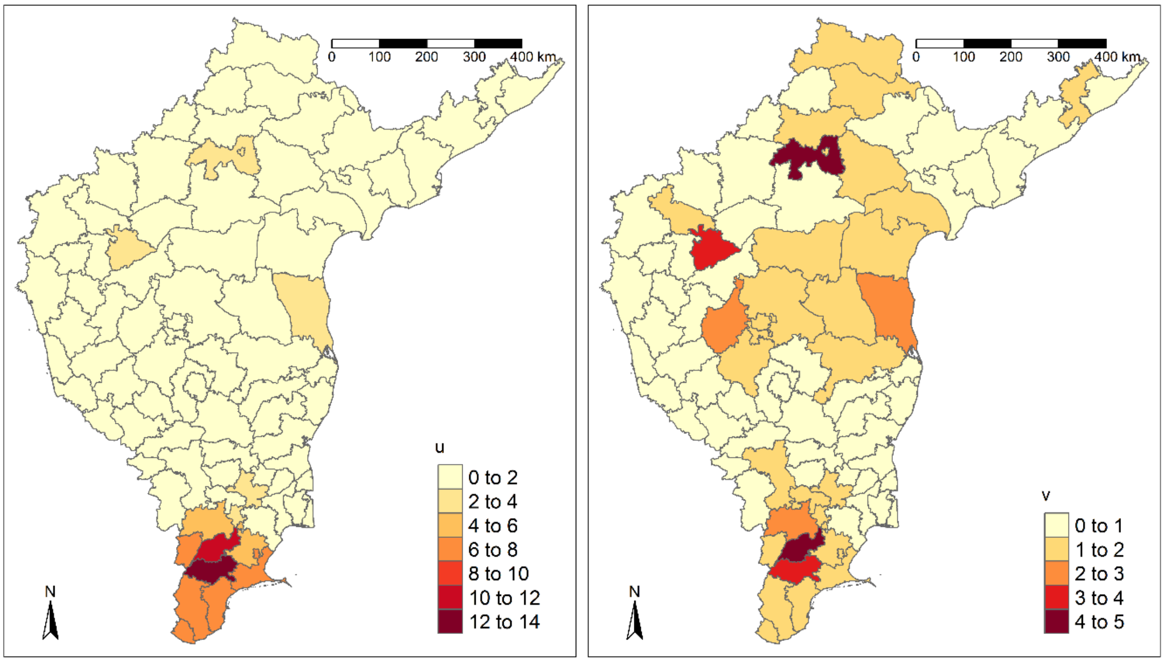


Fig. S7. District level values of spatial random effect components where u is the scaled spatially structured effect (left-hand panel) and v is the unstructured effect (right-hand panel). These maps may help in identifying environmental predictors that are missing from the model that match these random effects in spatial pattern and also indicate that these random effects values are particularly high in value for Tamil Nadu at the tip of India.

Table S3. Comparison of accuracy metrics for top performing host models fitted with spatially structured and unstructured random effects (bym2, as described in main text), models with spatially structured random effects only (besag) and non-spatial models (non-spatial) with no random effects.

(a) BYM2 model with spatially structured and unstructured random effects

| Fixed effects in the model | DIC | pD | mean deviance | Log score | RMSE | Pearson's r |
|---|---|---|---|---|---|---|
| bt ~ buffalos + nsheep + esheep + icattle | 197.6 | 45.6 | 152.7 | 1.64 | 0.66 | 0.997 |
| bt ~ buffalos + nsheep + esheep + icattle + goats | 197.9 | 46.1 | 152.6 | 1.70 | 0.64 | 0.997 |
| bt ~ buffalos + nsheep + esheep + icattle + cbcattle | 198.2 | 45.9 | 153.0 | 1.66 | 0.65 | 0.997 |
| bt ~ buffalos + nsheep + esheep + icattle + cbcattle + goats | 198.4 | 46.4 | 152.8 | 1.71 | 0.63 | 0.997 |
| bt ~ buffalos + nsheep + esheep | 198.5 | 45.5 | 153.8 | 1.63 | 0.66 | 0.997 |
| bt ~ buffalos + nsheep + esheep + goats | 198.8 | 45.9 | 153.6 | 1.67 | 0.64 | 0.997 |
| bt ~ buffalos + nsheep + esheep + cbcattle | 199.2 | 45.8 | 154.2 | 1.64 | 0.66 | 0.997 |
| bt ~ buffalos + nsheep + esheep + cbcattle + goats | 199.4 | 46.2 | 154.0 | 1.68 | 0.64 | 0.997 |
| bt ~ buffalos + nsheep + icattle | 199.6 | 46.1 | 154.2 | 1.65 | 0.66 | 0.997 |
| bt ~ buffalos + nsheep + icattle + goats | 199.6 | 46.4 | 154.0 | 1.71 | 0.64 | 0.998 |

(b) Besag model with only spatially structured random effects

| Fixed effects in the model | DIC | pD | mean deviance | Log score | RMSE | Pearson's r |
|---|---|---|---|---|---|---|
| bt ~ buffalos + nsheep + esheep + icattle | 200.1 | 46.3 | 154.7 | 1.65 | 0.67 | 0.997 |
| bt ~ buffalos + nsheep + esheep + icattle + goats | 200.4 | 46.5 | 154.7 | 1.70 | 0.66 | 0.997 |
| bt ~ buffalos + nsheep + esheep + icattle + cbcattle | 200.5 | 46.7 | 154.7 | 1.66 | 0.66 | 0.997 |
| bt ~ buffalos + nsheep + esheep + icattle + cbcattle + goats | 200.8 | 46.9 | 154.7 | 1.71 | 0.64 | 0.997 |
| bt ~ buffalos + nsheep + esheep | 200.4 | 46.0 | 155.2 | 1.64 | 0.68 | 0.997 |
| bt ~ buffalos + nsheep + esheep + goats | 200.6 | 46.2 | 155.2 | 1.68 | 0.66 | 0.997 |
| bt ~ buffalos + nsheep + esheep + cbcattle | 200.9 | 46.4 | 155.3 | 1.65 | 0.67 | 0.997 |
| bt ~ buffalos + nsheep + esheep + cbcattle + goats | 201.1 | 46.6 | 155.4 | 1.69 | 0.65 | 0.997 |
| bt ~ buffalos + nsheep + icattle | 202.6 | 47.3 | 156.0 | 1.69 | 0.66 | 0.997 |
| bt ~ buffalos + nsheep + icattle + goats | 202.6 | 47.3 | 156.1 | 1.75 | 0.65 | 0.997 |

(c) Non-spatial model – no spatial random effects

| Fixed effects in the model | DIC | pD | mean deviance | Log score | RMSE | Pearson's r |
|---|---|---|---|---|---|---|
| bt ~ buffalos + nsheep + esheep + icattle | 506.9 | 5.0 | 501.9 | 3.47 | 5.76 | 0.698 |
| bt ~ buffalos + nsheep + esheep + icattle + goats | 507.9 | 6.0 | 501.9 | 3.55 | 5.74 | 0.701 |
| bt ~ buffalos + nsheep + esheep + icattle + cbcattle | 508.8 | 6.0 | 502.8 | 3.52 | 5.78 | 0.696 |
| bt ~ buffalos + nsheep + esheep + icattle + cbcattle + goats | 509.7 | 7.0 | 502.8 | 3.60 | 5.76 | 0.699 |
| bt ~ buffalos + nsheep + esheep | 524.1 | 4.0 | 520.1 | 3.59 | 6.04 | 0.661 |
| bt ~ buffalos + nsheep + esheep + goats | 525.5 | 5.0 | 520.5 | 3.64 | 6.02 | 0.664 |
| bt ~ buffalos + nsheep + esheep + cbcattle | 525.0 | 5.0 | 520.0 | 3.64 | 6.11 | 0.653 |
| bt ~ buffalos + nsheep + esheep + cbcattle + goats | 526.1 | 6.0 | 520.1 | 3.69 | 6.08 | 0.656 |
| bt ~ buffalos + nsheep + icattle | 627.9 | 4.0 | 623.9 | 4.27 | 6.84 | 0.528 |
| bt ~ buffalos + nsheep + icattle + goats | 625.7 | 5.0 | 620.8 | 4.41 | 6.80 | 0.536 |

Table S4. Coefficient of variation for host predictors offered to the modelling process

| Predictor | Mean log density | s.d. | Coefficient of variation (%) |
|---|---|---|---|
| Indigenous cattle | 5.19 | 0.57 | 10.93 |
| Cross-bred cattle | 4.78 | 0.67 | 14.06 |
| Buffaloes | 4.98 | 0.71 | 14.34 |
| Non-descript sheep | 4.78 | 0.99 | 20.79 |
| Exotic sheep | 2.18 | 1.65 | 75.43 |
| Goats | 5.23 | 0.69 | 13.18 |

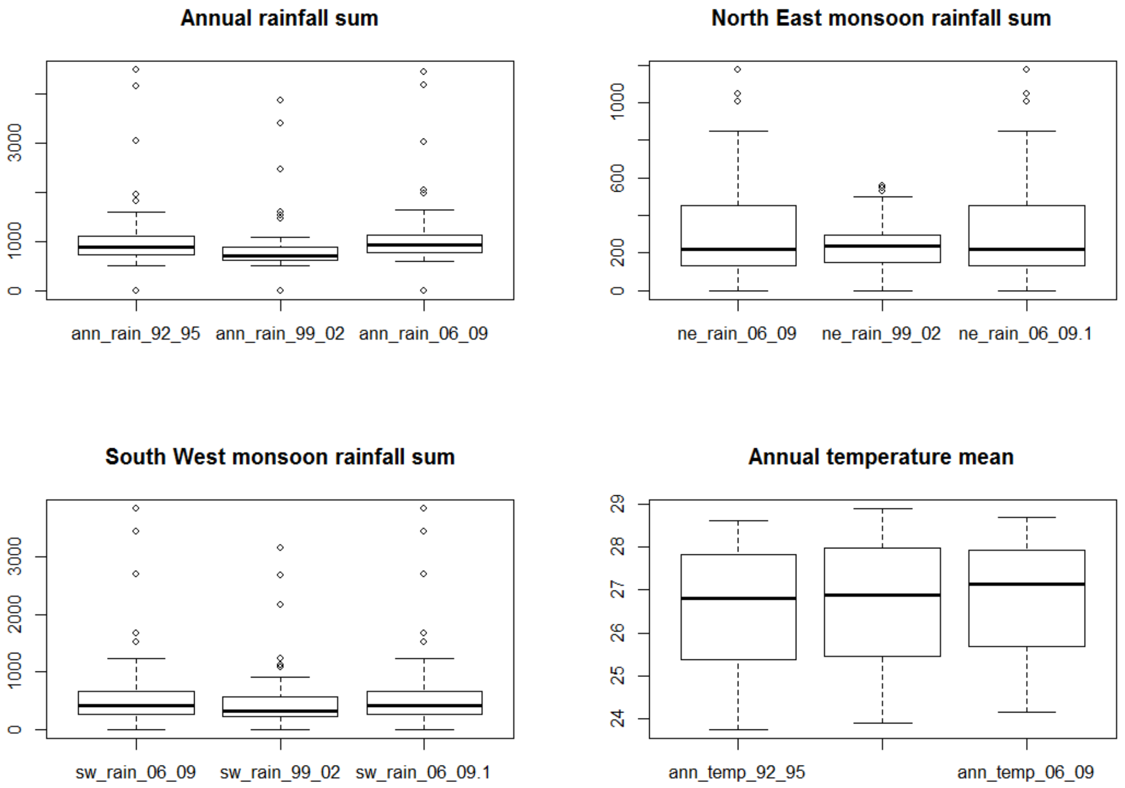


Fig. S8. Box plots indicating the variation in values of rainfall and temperature predictors across districts in South India between three different four-year periods across the study period. The heavy blacklines across the middle of the box indicate median values across districts, the box indicates the interquartile range of the data whilst the whiskers indicate the extremes. These plots indicate that there has been very little change in the median or range of values in the districts across the study period, though districts have become a fraction warmer from the start to the end of the period, and the middle period was drier than the start and end of the period.

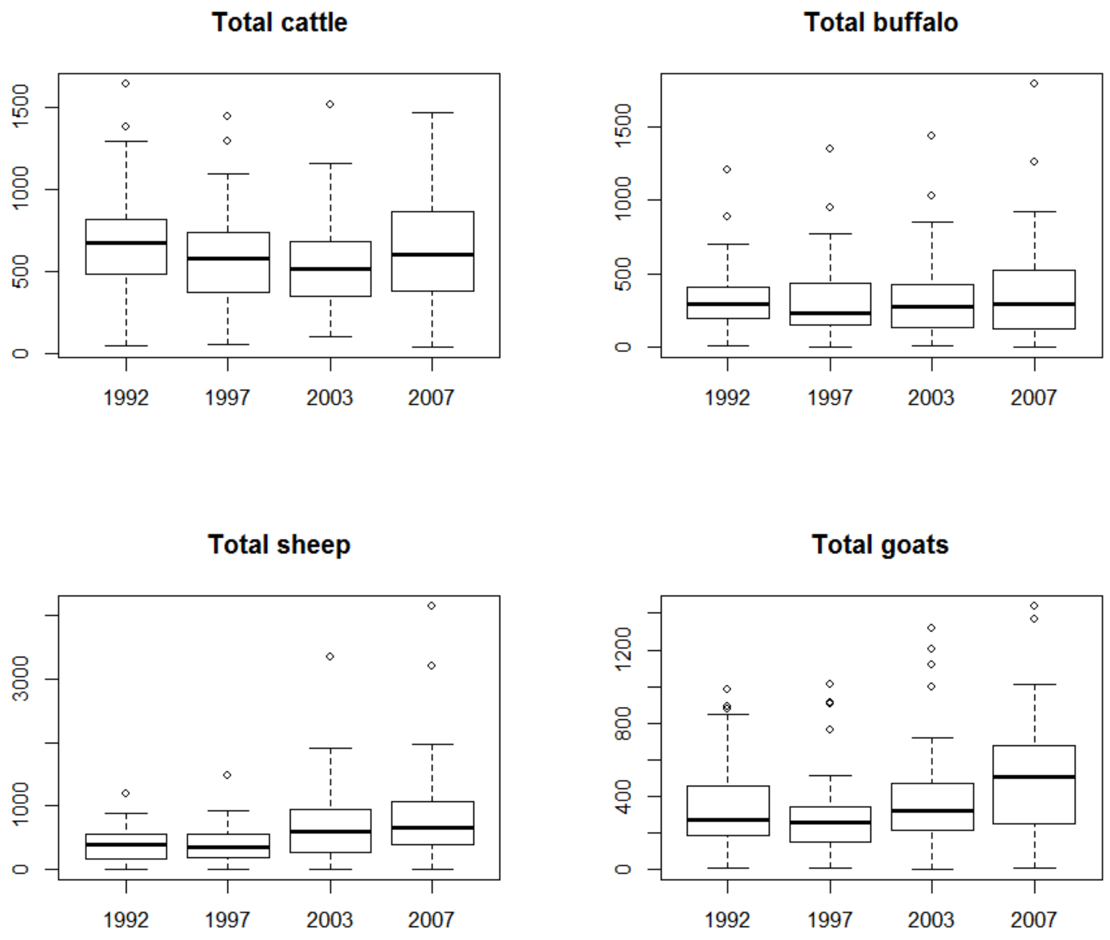


Fig. S9. Box plots indicating the variation in total numbers of different hosts in different livestock census periods for districts in South India (Data source: Department of Animal Husbandry, Dairying and Fisheries, Government of India). The heavy blacklines across the middle of the box indicate median values across districts, the box indicates the interquartile range of the data whilst the whiskers indicate the extremes. These plots indicate that the numbers of sheep and goats per district have increased over the study period, the number of cattle per district decreased before increasing again to values comparable to the start of the study period, whilst the number of buffalos per district remained fairly stable throughout the study period.

Table S5. Pearson's correlation coefficient values between rainfall and temperature predictors in different four-year periods for districts in South India. Values in a district are highly correlated between periods showing that wetter districts tend to stay wetter and warmer districts tend to stay warmer between periods. Data are from the Indian Meteorological Department.

(a) Period mean of annual rainfall sum

| | 1992-1995 | 1999-2002 | 2006-2009 |
|---|---|---|---|
| 1992-1995 | 1.000 | | |
| 1999-2002 | 0.979 | 1.000 | |
| 2006-2009 | 0.981 | 0.963 | 1.000 |

(b) Period mean of north east monsoon rainfall sum

| | 1992-1995 | 1999-2002 | 2006-2009 |
|---|---|---|---|
| 1992-1995 | 1.000 | | |
| 1999-2002 | 0.892 | 1.000 | 0.888 |
| 2006-2009 | 0.950 | 0.888 | 1.000 |

(c) Period mean of south west monsoon rainfall sum

| | 1992-1995 | 1999-2002 | 2006-2009 |
|---|---|---|---|
| 1992-1995 | 1.000 | | |
| 1999-2002 | 0.984 | 1.000 | |
| 2006-2009 | 0.985 | 0.989 | 1.000 |

(d) Period mean of annual mean temperatures

| | 1992-1995 | 1999-2002 | 2006-2009 |
|---|---|---|---|
| 1992-1995 | 1.000 | | |
| 1999-2002 | 0.998 | 1.000 | |
| 2006-2009 | 0.993 | 0.993 | 1.000 |

Table S6. Pearson's correlation coefficient values between host predictors in different census periods for districts in South India. Values in a district are highly correlated between livestock censi showing that districts with more of a particular livestock type at the start of the study period tend to have more of the same livestock type at the end of the study period.

(a) Census values for total number of cattle per district

| | 1992 | 1997 | 2003 | 2007 |
|---|---|---|---|---|
| 1992 | 1.000 | | | |
| 1997 | 0.885 | 1.000 | | |
| 2003 | 0.726 | 0.695 | 1.000 | |
| 2007 | 0.722 | 0.850 | 0.706 | 1.000 |

(b) Census values for total number of buffalos per district

| | 1992 | 1997 | 2003 | 2007 |
|---|---|---|---|---|
| 1992 | 1.000 | | | |
| 1997 | 0.970 | 1.000 | | |
| 2003 | 0.913 | 0.936 | 1.000 | |
| 2007 | 0.903 | 0.928 | 0.963 | 1.000 |

(c) Census values for total number of sheep per district

| | 1992 | 1997 | 2003 | 2007 |
|---|---|---|---|---|
| 1992 | 1.000 | | | |
| 1997 | 0.920 | 1.000 | | |
| 2003 | 0.725 | 0.875 | | |
| 2007 | 0.745 | 0.864 | 0.918 | 1.000 |

(d) Census values for total number of goats per district

| | 1992 | 1997 | 2003 | 2007 |
|---|---|---|---|---|
| 1992 | 1.000 | | | |
| 1997 | 0.841 | 1.000 | | |
| 2003 | 0.735 | 0.872 | 1.000 | |
| 2007 | 0.742 | 0.888 | 0.889 | 1.000 |
